# Mechanistic Insights and Design Strategies for Hydrogel/Aerogel Sorbents in Remediation of Per- and Polyfluoroalkyl Substances

**DOI:** 10.1021/acsenvironau.5c00081

**Published:** 2025-10-20

**Authors:** Ashvinder Kumar, Manju K. Thakur, Phil Hart, Vijay K. Thakur

**Affiliations:** † 3123 Biorefining and Advanced Materials Research Center, SRUC, Kings Buildings, West Mains Road, Edinburgh EH9 3JG, U.K.; ‡ Department of Chemistry, RNT Government College, Sarkaghat, District Mandi, Himachal Pradesh 175024, India; § The School of Water, Energy and Environment (SWEE), Cranfield University, Bedford MK43 0AL, U.K.; ∥ Renewable and Sustainable Energy Research Centre, 559361 Technology Innovation Institute, P.O. Box 9639, Abu Dhabi, United Arab Emirates

**Keywords:** PFAS, hydrogels, aerogels, fluorogels, thermosensitive hydrogels, adsorption, thermosensitive hydrogels, foams, water contamination

## Abstract

Per- and polyfluoroalkyl substances (PFAS) have been used for several decades in various sectors, including aerospace, construction, the military, and the production of goods, among others. This widespread use has significantly contaminated water bodies globally. Several government agencies and organizations are trying to develop advanced technologies such as oxidation, membrane filtration, adsorption, and ion-exchange resin to capture these chemicals and thus mitigate their impacts. Adsorption has proven to be a highly attractive method for removing PFAS, involving activated carbon, silica, bioadsorbents, anion-exchange resin, hydrogels, and nonion exchange polymers. Among different adsorbents, hydrogels are the most effective adsorbents for removing these forever chemicals due to their highly porous structure, reuse and regeneration ability, and ease of functionalization with specific groups for effective binding with PFAS molecules. Keeping in view their tremendous potential, this Review critically reviews the potential of underexplored hydrogel/aerogels-based sorbents developed from synthetic polymers as well as biopolymers. The use of different cross-linkers, co-monomers, inorganic and organic additives, and surface functionalization techniques on the PFAS removal ability of the resulting hydrogels/aerogels under varying pH, background species concentration, PFAS concentration, and temperature was thoroughly discussed. Furthermore, the underlying adsorption mechanisms (ionic, hydrophobic, hydrogen bonding, and F–F interactions) of hydrogels and aerogels for PFAS adsorption from a molecular perspective were also examined. Finally, the challenges inhibiting the large-scale production of these adsorbents and the scope of ionic fluorogel and thermosensitive hydrogels have also been thoroughly reviewed.

## Graphical abstract

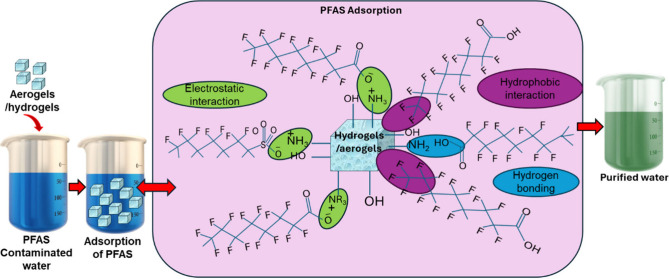

## 1 Introduction

Per- and polyfluoroalkyl substances (PFAS) are synthetic compounds with strong C–F bonds, valued for oil, water, stain, and soil repellence, thermal/chemical stability, and friction reduction, leading to extensive usage in the aerospace, automotive, textile, leather, construction, electronics, firefighting, food processing, and medical industries.^1,2^ Their environmental persistence and bioaccumulation have earned them the name “forever chemicals.” Structurally, PFAS are divided into polymers [fluoropolymers, perfluoropolyethers (PFPEs), side-chain fluorinated polymers] and nonpolymers [perfluoroalkyl iodides (PFAIs), ether-based PFAS, perfluoroalkane sulfonyl fluorides (PASF), and perfluoroalkyl acids (PFAAs)].^3^

These chemicals have been in use since 1950, but their presence in environmental samples was not widely documented until the early 2000s.^4^ Global emissions of C_4_–C_14_ perfluorinated carboxylic acids (PFCAs) between 1951–2015 were estimated at 2610–21,400 tons.^5^ PFAS have been detected in drinking water, landfill leachates, surface water, and coastal water. The U.S. Environmental Protection Agency (EPA) issued a Lifetime Health Advisory (LHA) in 2016 for perfluorooctanesulfonic acid (PFOS) and perfluorooctanoic acid (PFOA) at 70 ng/L,^6,7^ with subsequent findings showing widespread contamination in U.S. tap water^8,9^ and landfill leachates.^10^ In 2022, EPA revised LHA levels to 0.004 ng/L (PFOA) and 0.02 ng/L (PFOS), adding limits for hexafluoropropylene oxide (HFPO; 10 ng/L) and PFBS (2000 ng/L).^11^ In 2024, enforceable drinking limits were set at 4 ng/L (PFOA and PFOS) and 10 ng/L [perfluorononanoic acid (PFNA), HFPO and perfluorohexanesulfonic acid (PFHxS)].^12^

Other regions have set different limits, including the EU (<500 ng/L total PFAS, or <100 ng/L for 20 specific PFAS)^13^ and Canada (≤30 ng/L).^14^ Global testing found PFAS (sum of PFHxS, PFOS, PFOA, and PFNA) in rainwater exceeding these thresholds.^15^

Humans are exposed via PFAS-based products, contaminated food (fruits, fish, meat, and eggs), drinking water, and air. Long-chain PFAS (C > 6) are of greater concern due to persistence, low solubility, and protein binding.^9,16,17^ The Stockholm Convention now regulates PFAS, including long-chain PFCAs.^18^ Health impacts include liver toxicity,^19^ reproductive/developmental effects,^20^ neurotoxicity,^21^ immunotoxicity,^22^ and obesity.^23^ National Institute of Environmental Health Science (NIEHS)-funded research links PFAS to reduced bone density, delayed puberty, diabetes, thyroid/testicular cancer, and liver damage^24–28^ (Figure 1).

**Figure 1:**
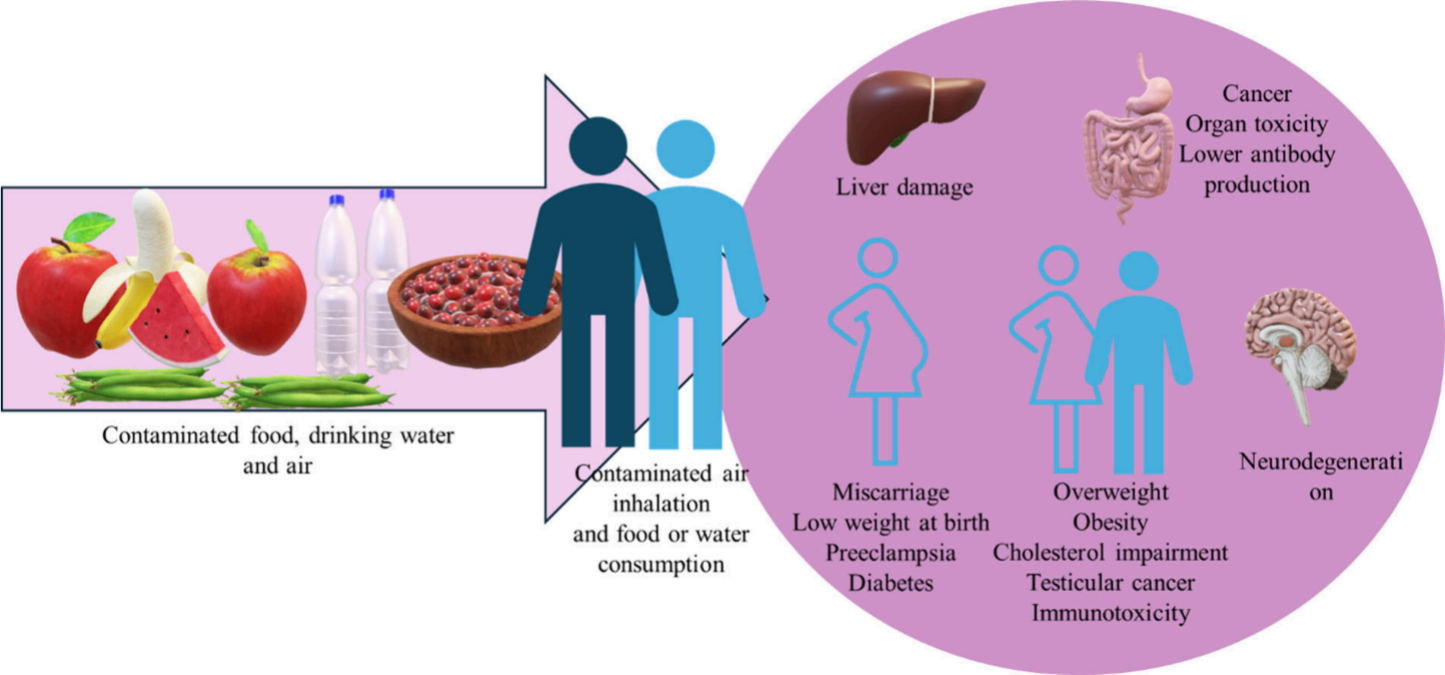
Effects of consumption of PFAS-contaminated water and food on the health of human beings.^28^ Figure adapted under Creative Commons Attribution International Licence (CC BY 4.0) from ref 28. Copyright 2022 Frontiers.

PFAS were first detected in exposed worker blood in the 1970s and later (1990) in the general population.^29^ Studies show long-chain PFAS (PFOA, PFOS, PFNA, PFHxS) are present in nearly (97%) all U.S. blood samples,^30,31^ though declines have been observed since 2000 due to phase-outs.^32^

The Stockholm Convention banned PFAS and their derivatives in 2009, PFOA in 2020, and PFHxS in 2022.^33^ The EU’s REACH regulation further restricts PFCAs and PFHxA in consumer goods.^34^ PFAS-based firefighting foams have been progressively restricted, with a full phase-out expected by 2025.^35^ The U.S. has proposed a federal ban on all nonessential PFAS within 10 years, phasing them out across industries.^36^ Japan restricts PFOS/PFOA and set a temporary water limit (50 ppt).^35^ New Zealand will ban PFAS in cosmetics by 2027, and Taiwan is developing drinking-water limits.^37^ Overall, regulations are increasing but remain uneven globally.

Conventional wastewater treatment plants (WWTPs) are ineffective for PFAS.^38–40^ Current remediation techniques include (Figure 2) the following: (1) physical methods such as adsorption and membrane filtration [reverse osmosis (RO), nanofiltration (NF)] achieve ∼95–97% removal^41,42^ but are limited by cost and fouling;^39,43^ (2) advanced oxidation processes (AOPs) such as light-induced advanced oxidation,^44^ nanomaterial-based advanced oxidation/reduction,^45^ UV photocatalysis,^46^ UV/H_2_O_2_,^47^ electrochemical oxidation,^48^ sonochemical methods,^49^ and hydrothermal/supercritical oxidation^50^ degrade PFAS but are energy-intensive and produce byproducts; (3) biodegradation such as microbial PFAS breakdown is promising^51–54^ but limited by strong C–F bonds and fluoride release;^55,56^ and (4) adsorption, which is low-cost, efficient, and widely applied. Adsorbents include carbon-based (biochar, AC, CNTs, graphene),^57–59^ zeolites,^60^ polymers,^61–63^ natural materials (lignin, chitosan, rice husk),^64,65^ metal–organic frameworks (MOFs),^66–68^ nanoparticles,^69^ hydrogels,^70,71^ and composites.^72^

**Figure 2:**
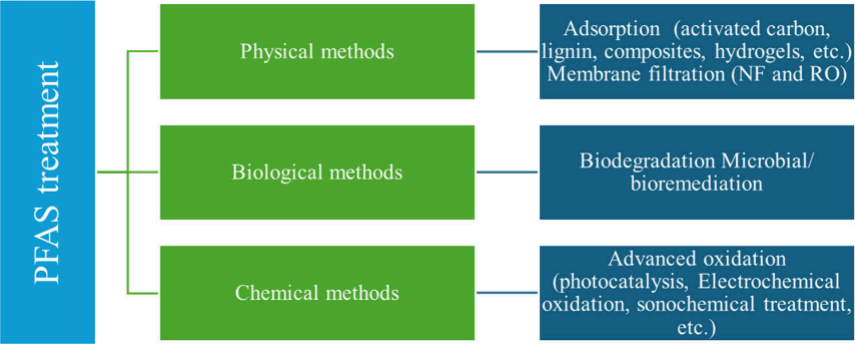
Different advanced techniques can be utilized for PFAS removal from contaminated water.

Hydrogels/aerogels, with high porosity and surface area and tunable chemistry, are emerging as promising PFAS adsorbents.^72^ Their efficiency depends on hydrophobic and electrostatic interactions, surface charge, PFAS structure, pH, and ionic strength.^43,71^ Unlike previous reviews on adsorbents,^61,73,74^ this work focuses on hydrogels/aerogels as potential materials for PFAS remediation (Figure 3).

**Figure 3:**
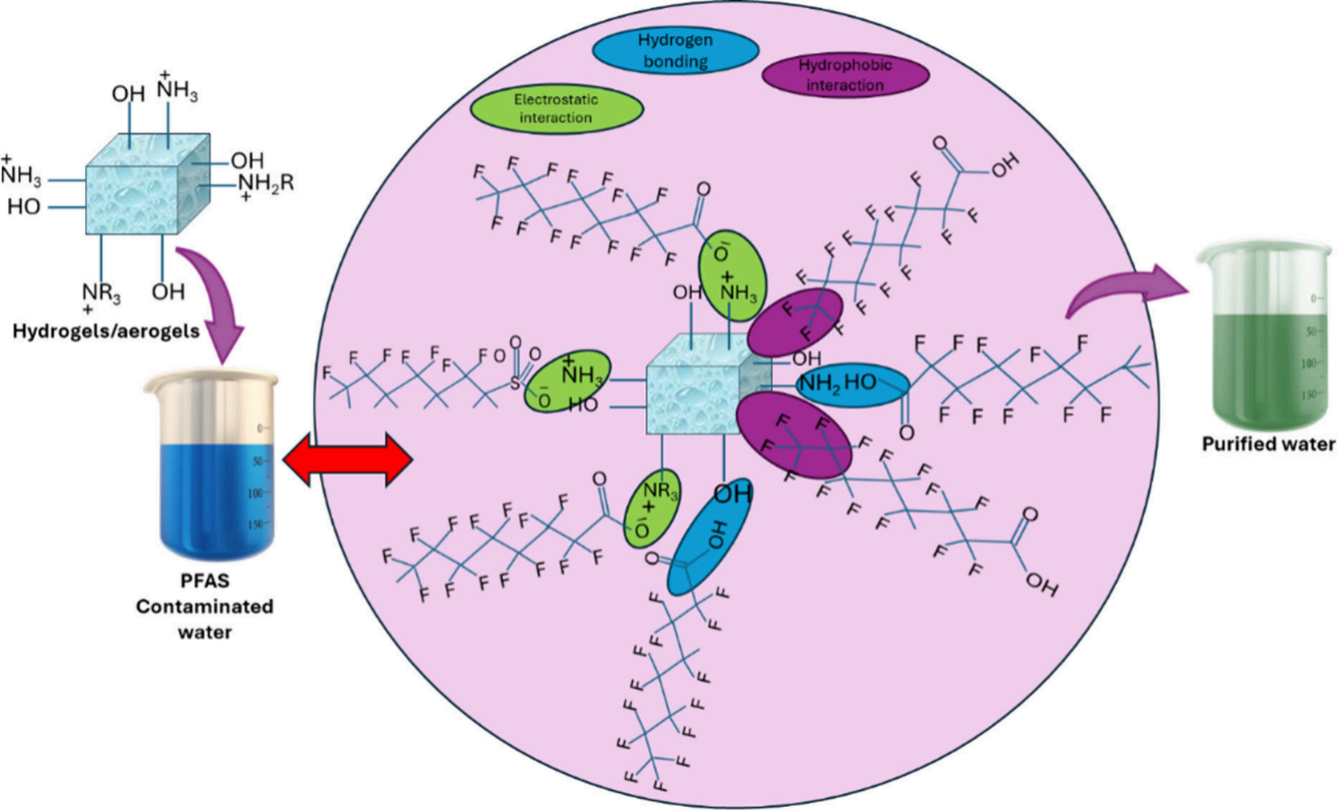
Possible mechanism for the interaction between hydrogels/aerogels and PFAS molecules.

## 2 PFAS Categories/Nomenclature, Different Sources, And Environmental Exposure

PFAS are aliphatic compounds in which hydrogen atoms are replaced by fluorine, partially (polyfluorinated) or fully (perfluorinated). The strong, highly polar and inert C–F bonds confer remarkable chemical and thermal stability, making PFAS highly persistent in water, soil, and air.^75,76^

They [general formula: CnF_2*n*+1_ (*n* ≥ 1)] are broadly categorized as long-chain (≥C6–C7) and short-chain (≤C6) species.^77^ Nonpolymeric long-chain PFAS include perfluoroalkyl acids including carboxylic acids (PFCAs, CnF_2*n*+1_­COOH, *n* ≥ 7), phosphonic acids (PFPAs, CnF_2*n*+1_­PO_3_H_2_, *n* ≥ 6), phosphinic acids (PFPiAs, CnF_2*n*+1_­PO_2_H, *n* ≥ 6), and sulfonic acids (PFSAs, CnF_2*n*+1_­SO_3_H, *n* ≥ 6), as well as PASF (CnF_2*n*+1_­SO_2_–R, *n* ≥ 6) and fluorotelomer iodides (FTIs; CnF_2*n*+1_­CH_2_CH_2_I, *n* ≥ 6). Short-chain members (CnF_2*n*+1_–R, *n* ≤ 6, with R = COOH or SO_3_H) include PFBA, PFBS, PFHxA, and PFHxS.^17^ PFOS (C_8_F_17_SO_3_H) and PFOA (C_7_F_15_COOH) are the most extensively used and globally detected, present in firefighting foams, cookware, food packaging, textiles, and cosmetics.^29^

Polymeric PFAS such as fluoropolymers [poly­(vinyl fluoride) (PVF), fluorinated ethylene propylene (FEP), polytetrafluoroethylene (PTFE), perfluoroalkoxy (PFA)], perfluoropolyethers, and side-chain fluorinated polymers are generally considered less bioactive, yet their large-scale manufacture has added significantly to environmental burdens. Consequently, regulation has focused on nonpolymeric PFAS, which bioaccumulate and exhibit adverse health effects.^76,78^ Their unique repellency, durability, and chemical resistance underpin over 200 applications and >1,400 individual products^79^ (Figure S1). Figure 4 shows a hypothesized life cycle of PFAS.

**Figure 4:**
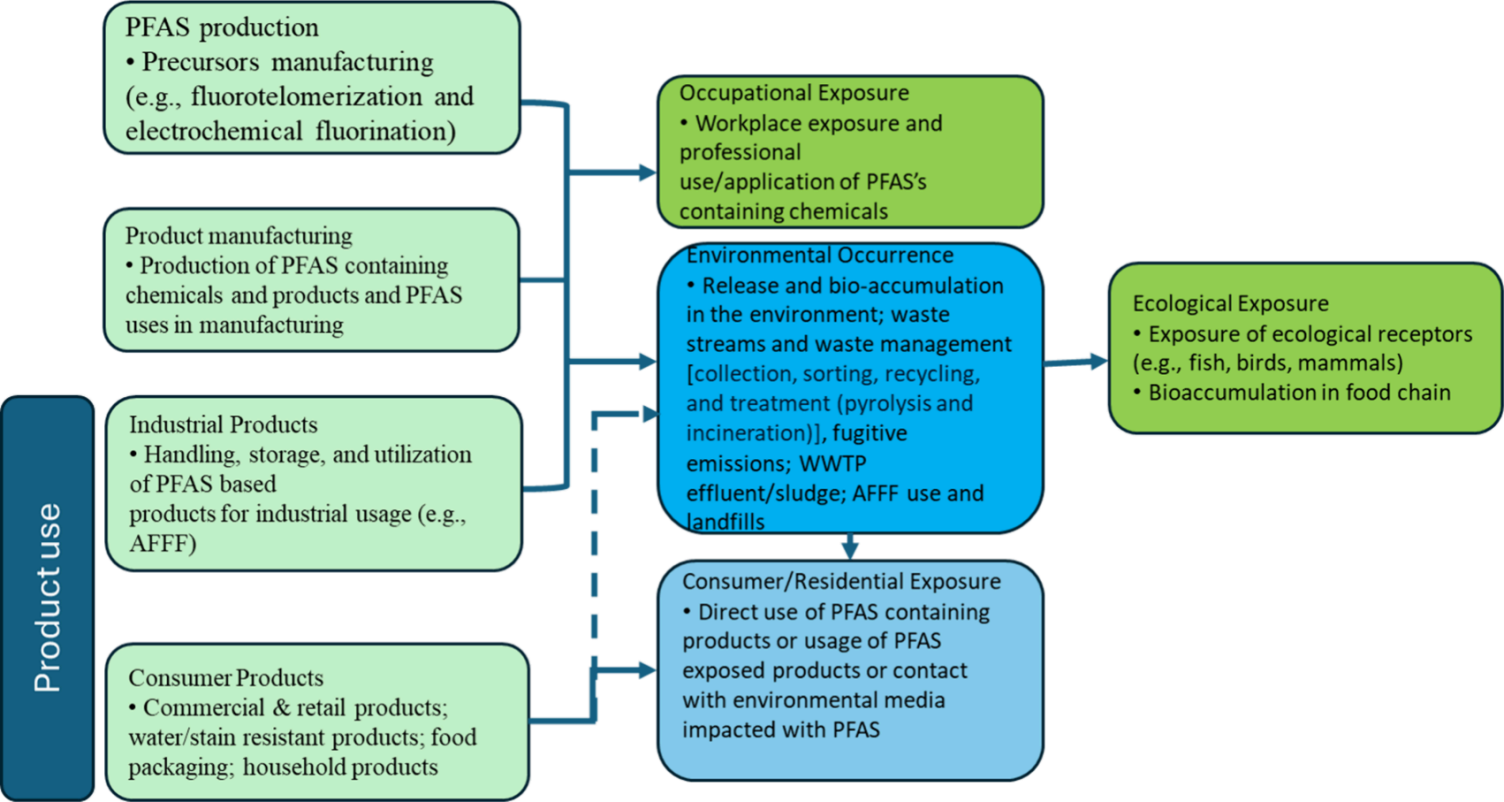
PFAS utilization and potential for human exposure and environmental contamination during the PFAS life cycle.^81^ Adapted under CC BY 4.0 from ref 81. Copyright 2020 ITRC.

PFAS during manufacture and disposal contaminate soil, air, and aquatic systems through air emissions, spills, and wastewater discharges.^80^ WWTPs effluents, firefighting foams, and landfill leachates remain dominant contributors to environmental pollution.^81^

## 3 Role of Hydrogels/Aerogels in PFAS Removal

Hydrogels possess large surface area, high porosity, strong hydrophilic–hydrophobic interactions, exceptional swelling capacity, and chemical stability, making them attractive for PFAS remediation.^62,71^ These cross-linked polymer networks imbibe large amounts of water without dissolving due to embedded hydrophilic groups.

Depending on composition (natural, synthetic, or hybrid) and application, hydrogels are cross-linked chemically (covalent bonds) or physically (hydrogen bonding, hydrophobic, or ionic interactions).^82–84^ Chemical cross-linking typically yields greater robustness and thermal stability.^82,85^ Postprocessing (e.g., freeze-drying, supercritical drying) produces aerogels, foams, or membranes with enhanced porosity, surface area, and strength, which are critical for PFAS adsorption.^86^ A detailed description of the potential of different hydrogels, aerogels, foams, etc., for the removal of PFAS has been given in the current section (Table 1).

**Table 1:** Comparative View of the PFAS Adsorption Capacity of Different Hydrogels/Aerogels/Foams

**Adsorbent**	**Total surface area (m^2^/g)**	**Dose (mg/L)**	**PFAS**	**C0 (mg/L)**	**Time (h)**	**Sorption capacity**	**Ref**
**T25M70A5**	582.76 ± 7.13	2 g/L	PFOA and PFOS	5–200 mg/L	24	PFOA: 61.8–98.0% removal efficiency; PFOS: 94.5–100.0%	^99^
**ALGPEI-3 aerogel**	1.02	100 mg/L	PFDA, PFHxA, PFOS, PFHpA, PFOA, PFHxS, PFNA, PFBS, PFUnA, GenX, 6:2 FTSA and 2N-EtFOSAA	0.01–0.5	24	3045.28 mg/g	^122^
**GTH–CSPEI aerogel**	3.79	100 mg/L	PFDA, PFHxA, PFOS, PFHpA, PFOA, PFHxS, PFNA, PFBS, PFUnA, GenX, 6:2 FTSA and 2N-EtFOSAA	0.01–0.5	24	12133.14 mg/g	^122^
**A-PEGDA**	--	30 mg/L	PFOA	1	12	109.9 ± 8.5 μmol/g	^115^
			PFOS			30.4 ± 0.4 μmol/g	
			PFBA			199.5 ± 10.8 μmol/g	
			PFBS			190.6 ± 21.0 μmol/g	
			Gen X			86.7 ± 5.1 μmol/g	
**F-PEGDA**	--	30 mg/L	PFOA	1	12	21.3 ± 0.2 μmol/g	^115^
			PFOS			2.2 ± 0.4 μmol/g	
			PFBA			24.0 ± 7.1 μmol/g	
			PFBS			0	
			Gen X			0	
**AF-PEGDA**	--	30	PFOA	1	12	110.6 ± 9.7 μmol/g	^115^
			PFOS			30.4 ± 2.2 μmol/g	
			PFBA			158.0 ± 0.5 μmol/g	
			PFBS			168.9 ± 10.7 μmol/g	
			Gen X			98.7 ± 3.9 μmol/g	
**AGO aerogels**	--	1	PFOA	20 to 1300	48	1575 mg/g	^107^
**5N5P**	--	0.5 g/L	PFOA and PFOS	100 μg/L each	24	96% of PFOA; 98% of PFOS	^70^
**Cu/F-rGA aerogel**	133.49	6	PFOA	2	12	17.86 mg/g	^100^
**Cu-rGA aerogel**	151.65	6	PFOA	2	12	13.72 mg/g	^100^
**F-rGA aerogel**	203.0	6	PFOA	2	12	6.24 mg/g	^100^
**3D-SHΔ** *a* **erogel**	140 mm2	1.5 g/L	PFOA	5 to 200 ppm	24	33.55 mg/g	^71^
**3D-PSHΔaerogel**	540 mm2	50 g/L	PFOA	5 to 200 ppm	24	51.3 mg/g	^71^
**IF-20+**	--	100 mg/L	GenX	0.20 to 50 mg/L	3	278 mg/g	^117^
**IF-1**	--	100 mg/L	GenX	0.20 to 50 mg/L	3	280 mg/g	^118^
**3D SG-TiO**_**2**_ **QDa**	--	20 mg/L	PFOA	0.5 to 20 mg/L	4	0.0632 mmol/g	^90^
			PFHpA	0.5 to 20 mg/L		0.05773 mmol/g	
			PFHxA	0.5 to 20 mg/L		0.05634 mmol/g	
			PFPeA	0.5 to 20 mg/L		0.05033 mmol/g	
**PAM–PANI-2**	17.853	1 g/L	PFOA	1 μg/L	15 min	41.7 mg/g	^116^
**PNIPAM/PMMA/CS-3**	--	0.2 g/L	PFOS	500 mg/L	24 h	476.5 mg/g	^130^
**CTAB-functionalized alginate hydrogel**	15	0.25 g/L	PFOA	50 mg/L	24 h	382.1 mg/g	^126^
**CD_66_–0.2 PEG/PPG**	--	50 mg/L	PFOS	275 mg/L	48 h	2.84 g/g	^109^
**CEGH**	--	100 mg/L	PFOA	900 mg/L	24 h	1275.9 mg/g	^110^
**PNIPAM**	--	0.5 g/L	PFOA	50 mg/L	24 h	3.5 mg/g	^131^
**FC4**	--	--	PFOA	150 mg/L	2 h	218.4 mg/g	^108^
**SA-β-CDEX**	--	1280 mg	PFOS	10 mL of 10.0 ppm	30 min	0.0764 mg/g	^127^
**GCBs**	0.474 ± 0.13	220 mg/L	PFOS	100 mg/L	24 h	500 μmol/g	^129^
			PFOA			555.5 μmol/g	
			PFBS			769.2 μmol/g	
			PFBA			1428.6 μmol/g	
**ECH–CSPEI aerogel**	--	500 mg/L	11 PFAS	100 ng/L of each	48 h	PFHxA: 0.19; PFHpA: 0.11; PFOA: 0.19; PFNA: 0.18; PFDA: 0.14; PFBS: 0.17; PFHxS: 0.12; PFOS: 0.12; GenX: 0.09; 6:2 FTS: 0.1; N-EtFOSAA: 0.1 ng/mg	^123^

Synthetic hydrogels,^87^ derived from man-made precursors, and hybrid hydrogels,^88,89^ which integrate biomaterials or inorganic fillers with synthetic monomers, both demonstrate significant potential in removal of PFAS from contaminated water. Their performance can be tuned by surface functionalization or by incorporating additives such as photocatalysts,^90^ AC,^70^ or advanced oxidizing materials,^91^ thereby coupling adsorption with degradation pathways. Amine-functionalized and chitosan- and polyaniline-based hydrogels have performed well in batch and flow systems.^92^

PFAS capture occurs through multiple mechanisms (adsorption, ionic interaction, oxidation, and photochemical degradation) when used as a support material in hybrid filtration systems. Electrostatic attraction is dominant when negatively charged PFAS interact with cationic groups such as amines or quaternary ammonium functionalities. Hydrophobic interactions favor the adsorption of long-chain PFAS, while polar head groups such as sulfonates and carboxylates can form hydrogen bonds with amide-, amine-, or hydroxyl-containing hydrogel networks. Fluorinated coatings can enhance affinity toward long-chain PFAS, whereas ionic functionalities improve the uptake of short-chain analogues.

Hydrogels and their derivatives thus offer versatile platforms for PFAS remediation, benefiting from inherent hydrophilicity, biodegradability, and ease of modification. Yet critical challenges remain: adsorption efficiency, selectivity across diverse PFAS chemistries, regeneration capacity, and scalability under field conditions. Addressing these limitations through rational material design and hybrid system integration is essential for translating hydrogel-based technologies from proof-of-concept demonstrations to practical and sustainable solutions for PFAS-contaminated water.

### 3.1 Inorganic Hybrid Hydrogels/Aerogels

Inorganic–polymer hydrogels have recently gained prominence owing to the synergistic properties of inorganic fillers and polymer networks.^93,94^ Fillers such as silica, carbonaceous materials (AC, graphene oxide, and carbon dots), transition metal oxides, nanoparticles, and carbon nanotubes enhance mechanical strength, surface area, porosity, adsorption capacity, and catalytic stability, thereby improving PFAS remediation.^95^ Optimizing filler type and content tailors hydrogels for adsorption, ion exchange, or degradation, while derivatives like foams and aerogels further expand their functionality and utility in PFAS removal.

#### 3.1.1 Inorganic Materials like Metal Oxides, Metal Nanoparticles, and Silica-Based Hydrogels/Aerogels

Transition metals, their oxides, and silica have been widely used as fillers to prepare hydrogels that adsorb PFAS. These inorganic materials may significantly enhance the mechanical toughness, thermal strength, functionalization, and swelling behavior of the hydrogel matrix and were previously utilized as fillers in various applications, such as tissue engineering, drug delivery, sensors, and environmental remediation.^96–98^

Roque et al.^99^ designed three silica-based aerogels via the sol–gel method using varying compositions [T25M70A5 (TEOS, MTMS and APTES: 25% mol Si, 70% mol Si and 5% mol Si, respectively); T30M70; T10M85A5; and T20M70A10] of tetraethyl orthosilicate (TEOS), methyltrimethoxysilane (MTMS), and 3-aminopropyltriethoxysilane (APTES) through oven drying, freeze-drying and CO_2_ supercritical drying techniques to adsorb PFOA, PFOS, and PFBS. Among all samples, the T25M70A5 aerogel obtained through the freeze-drying technique exhibited the best results, with impressive removal efficiencies of 94.5–100.0% and 61.8–98.0% for PFOS and PFOA, respectively, at PFAS concentrations ranging from 5 to 200 mg/L. Furthermore, the T25M70A5 and T30M70 samples exhibited water contact angles ranging from 80° to 90°, indicating a good balance between hydrophobic and hydrophilic characteristics. Liu et al.^100^ utilized the microbubble template method to prepare Cu nanoparticles and a fluorine-modified graphene aerogel (Cu/F-rGA) for efficient PFOA adsorption. Their methodology includes doping the F element into the GO in the first step, followed by its conversion into 3D aerogel (F-rGA) and finally loading Cu NPs onto the F-rGA. For comparative purposes, they also prepared rGA and Cu NP-loaded rGA. Benefiting from hydrophobic and F–F attraction and the ligand exchange reaction, Cu/F-rGA (0.7813 mg/g min) was noted to have a 2.68 times higher initial PFOA adsorption rate in comparison to unmodified aerogels (rGA; 0.2915 mg/g min). Further, the adsorption capacity of Cu/F-rGA (17.86 mg/g) was also noted to be significantly higher than its counterparts, namely, GO (1.51 mg/g), rGA (3.76 mg/g), F-rGA (6.24 mg/g), and Cu-rGA (13.72 mg/g), at a 2 mg/L equilibrium concentration of PFOA. The adsorption was observed to follow pseudo-second-order kinetics, confirming the existence of both chemical and physical adsorption of PFOA onto the aerogels. Further, out of three adsorption isotherm models, i.e., Freundlich, Langmuir, and statistical physics models (1–3), Model 3 was noted to be the best fit, defining adsorption of the adsorbate via a variable number of layers. It was observed that when the temperature rose from 20 to 40 °C, the density of receptor sites and adsorption layers on adsorbents decreased from 25.51 to 12.54 mg/g and from 1.63 to 2.51, respectively. The Cu/FrGA retained 73.26% regeneration ability with ethanol after 10 adsorption–desorption cycles.

TiO_2_ quantum dot (TiO_2_) loaded (2–3 nm) sulfonated graphene (SG) 3D aerogels (3D SG-TiO_2_ QDs), for the simultaneous adsorption and decomposition of four PFAS, namely PFOA, perfluorohexanoic acid (PFHxA), perfluoroheptanoic acid (PFHpA), and perfluoro-n-pentanoic acid (PFPeA), were fabricated from predeveloped SG-TiO_2_ QD nanosheets [worked as building blocks; synthesized using varied concentrations of sodium dodecyl sulfate (1, 0.5 and 0 mol/L), along with TiCl_3_ and SG].^90^ The aerogels exhibited remarkable PFAS adsorption and photocatalytic decomposition abilities, resulting from the synergistic effect of TiO_2_ QDs (photocatalytic activity) and 3D SG (adsorption ability). Among different aerogels, the SG-TiO_2_ QDa (aerogels synthesized using 1 mol/L of SDS) showed better adsorption ability toward all the PFAS than TiO_2_ QDb (made by 0.5 mol/L of SDS) and TiO_2_ QD. The adsorption matched to the Langmuir adsorption model, indicating monolayer adsorption rather than multilayer adsorption. Further, during the photocatalytic photodegradation of PFOA, 3D SG-TiO_2_ QDa showed the fastest kinetics *k*_app_ (1.898 × 10^–4^/s), followed by 3D SG-TiO_2_ QDb (1.530 ×10^–4^/s) and 3D SG-TiO_2_ NP (9.283 × 10^–5^/s). Contrary to the above findings, Hwang et al.,^101^ while evaluating the potential of Ag/Au-loaded poly­(acrylic acid)/poly­(allylamine) hydrochloride hydrogel fibers to remove PFOS and PFOA, reported that it is the electrochemical effect (electrochemical oxidation of PFOA and PFOS), not the adsorption factor, that plays a crucial role in PFAS removal.

#### 3.1.2 Carbon-Based Hybrid Hydrogels/Aerogels

Incorporating carbonaceous components such as AC, graphene oxide (GO), and carbon dots (CDs) into hydrogels improves adsorption by providing high surface area, biocompatibility, and tailorable surface chemistry. AC, widely used for decades, is produced by pyrolyzing and activating biomaterials^94^ (e.g., wood, bamboo, nutshells; activated at high temperatures through chemical or steam treatment^102^) to form a porous network and is applied either as powdered (<80 μm, for rapid adsorption) or granular form (0.2–5 μm, for large-scale treatment).^94,103^ GO, synthesized from graphene via the Hummers method, offers a sheet-like structure with abundant oxygen-containing groups. CDs, typically 1–10 nm in size, provide high porosity and emerging potential as fillers for PFAS adsorption.^104–106^ Across these materials, functional groups such as carbonyl, carboxyl, hydroxyl, ether, and amino enhance hydrophobic, electrostatic, and hydrogen bonding interactions, thereby boosting PFAS uptake.

Tian et al.^107^ prepared amino-functionalized graphene oxide (AGO) aerogels by treating graphene oxide, extracted from graphene powder through Hammer’s method, with ethylene diamine, followed by freeze-drying, and subsequently used the aerogels for PFOA removal from contaminated water. Various parameters like time (varied from 0.5 to 48 h), temperature (298, 308, 318 K), solution pH (varied from 1.6 to 9.3), and absorbent amount (1 to 10 mg) were optimized to attain maximum removal of PFOA from 10 or 1000 mg/L concentrated solution. The GO after functionalization exhibited an increase in adsorption capacity, showing a maximum removal of 99.95% and adsorption capacity of 1575 mg/g for PFOA in a solution containing 10 and 1000 mg/L of adsorbate, respectively, at pH 3, a temperature of 298 K, a time of 48 h, and sorbent amounts of 10 and 1 mg, respectively. This increase in their adsorption ability has been attributed to the presence of amino groups and interconnected highly porous microstructures after amine functionalization. Further, the adsorption was found to follow pseudo-second-order kinetics and the Freundlich isotherm modeling, validating the multilayer adsorption of PFOA on a heterogeneous aerogel surface. Five different types of GO-based hydrogels, FC1, FC2, FC3, FC4, and FC5, for simultaneous oxidation and adsorption of PFOA were developed by reacting GO with FeSO_4_·7H_2_O, FeSO_4_·7H_2_O + ethylene glycol (EG), FeSO_4_·7H_2_O + EG + NaClO, FeSO_4_·7H_2_O + EG + ascorbic acid (AA), and EG + AA, respectively.^108^ FC4 hydrogels were noted to have the lowest degree of agglomeration and maximum porosity. The types of iron crystals in FC5 and FC1, FC3 and FC2, and FC4 were found to be α-FeOOH, Fe_3_O_4_, α-FeOOH, and Na_3_FeO_4_, and Fe_3_O_4_, respectively. Among the five hydrogels, FC4 exhibited the highest tendency for PFOA removal (89.9%) through the Fenton reaction, followed by FC3 (84.35%), FC5 (75.44%), FC2 (75.2%), and FC1 (73.61%). Like Fenton oxidation results, the removal of PFOA through adsorption was also found to be maximum in the case of FC4 (41.5%) and reached an equilibrium state within 120 min. Furthermore, using the Langmuir isotherm model, FC4 was calculated to have a maximum adsorption capacity of 218.4 mg/g for PFOA. The hydrogels exhibited excellent PFOA removal efficiency in a wide pH range (found maximum at pH 8 for FC4) and retained their removal rate even after 5 cycles. The collective rate of PFOA removal using FC4 was reported to be 89.9%, with adsorption accounting for approximately 41.5%.

Klaus et al.,^70^ prepared powdered porous activated carbon (PAC)-acrylamide-based hydrogel composites with varying relative compositions of *N*,*N*′-methylenebis­(acrylamide) (NNMBA) and AAm (9:1, 95:5, and 90:10 mol %) and PAC composition (1 and 5 mass %) and subsequently used them for the removal of PFOS and PFOA from a solution of 100 μg/mL concentration of each. The 5N5P, i.e., the hydrogel with 5% PAC and 95:5 relative proportions of AAm:NNMBA, when immersed in PFAS solution at a dosage of 0.5 mg/mL, pH 7.0, and temperature of 25 °C for 24 h, exhibited maximum removal efficiencies of 97.51% and 96% for PFOS and PFOA, respectively. This was followed by 1N5P (containing 1% mass of PAC instead of 5%; all other precursors were at the same amount), which showed 95.65% and 86.87% removal efficiencies for PFOS and PFOA, respectively. However, PAC outperformed the hydrogel, demonstrating removal abilities of 100% and 96% against PFOS and PFOA, respectively. Lee et al.^91^ used a carbon aerogel (CA) thermally activated persulfate system (PS) for the removal (advanced oxidation combined with adsorption) of PFOA, which was carried out at three different temperatures, i.e., at 25, 40, 50, and 60 °C. The CA + PS showed a removal efficiency of 85.7% after 8 h at 60 °C, much higher than PS (58.2%) and CA (14.5%) individually, due to the catalytic effect on the CA + PS surface, which activates PS for the generation of SO_4_^●–^ to enhance the decomposition of PFOA.

Wang et al.^109^ prepared highly efficient carbonaceous adsorbent material [carbon dots (CD) hydrogels] by cross-linking the zero-dimensional and nanosized amine group-capped carbon dots (CDs; content varied from 28–66%) with polypropylene glycol diglycidyl ether (PPG) and polyethylene glycol diglycidyl ether (PEG) (Figure 5). Among different CD-based hydrogels, CD_66_-0.2PEG/PPG (containing 66% of CDs contents; and a 0.2 ratio of PEG with respect to PPG) showed a maximum absorption capacity of 2.82 g/g (recorded at pH 7) for PFOS, which is higher than other carbonaceous materials reported to date (Figure 5b). Through the determination of the point of zero charge (pHpzc; noted to be a little higher than 9), it has been confirmed that at pH 11 the amine group remains in a neutral state and thus loses its ability to absorb PFOS. However, at pH 9 or a lower value, it works well. When used to adsorb short-chain PFASs, namely PFHxS and PFBS, a decrease in adsorption capacity with a reduction in chain length are noted, indicating the higher adsorption ability of hydrogels toward longer-chain PFASs (Figure 5c). Furthermore, the CD hydrogels followed pseudo-second-order kinetics during the adsorption of PFAS, indicating the dominance of the chemisorption; in this case, the rate of adsorption was found to be proportional to the square of the number of vacant sites. Additionally, during the adsorption isotherm experiment, the CD-based hydrogel exhibited a Langmuir adsorption mechanism, confirming homogeneous adsorbent surfaces with identical sorption sites (Figure 5d). The hydrogels maintained good adsorption ability after five adsorption–desorption cycles (regenerated by methanol washing) and efficiently treated neutral fire-fighting wastewaters containing higher concentrations of PFOS. A novel chitosan-ethylene glycol hydrogel (CEGH) was synthesized utilizing ethylene glycol and chitosan through a repeated freezing–thawing technique and subsequently used to remove PFOA.^110^ The adsorption was found to be best fit to the pseudo-second-order kinetics and the Freundlich–Langmuir isotherm model, showing a maximum adsorption ability of 1275.9 mg/g. Adsorption was found to increase from 20 to 40 °C and decrease when pH increased from 2 to 10. Ionic hydrogen bond interactions between carbonyl groups of PFOA and protonated amines were confirmed to be the primary removal mechanism.

**Figure 5:**
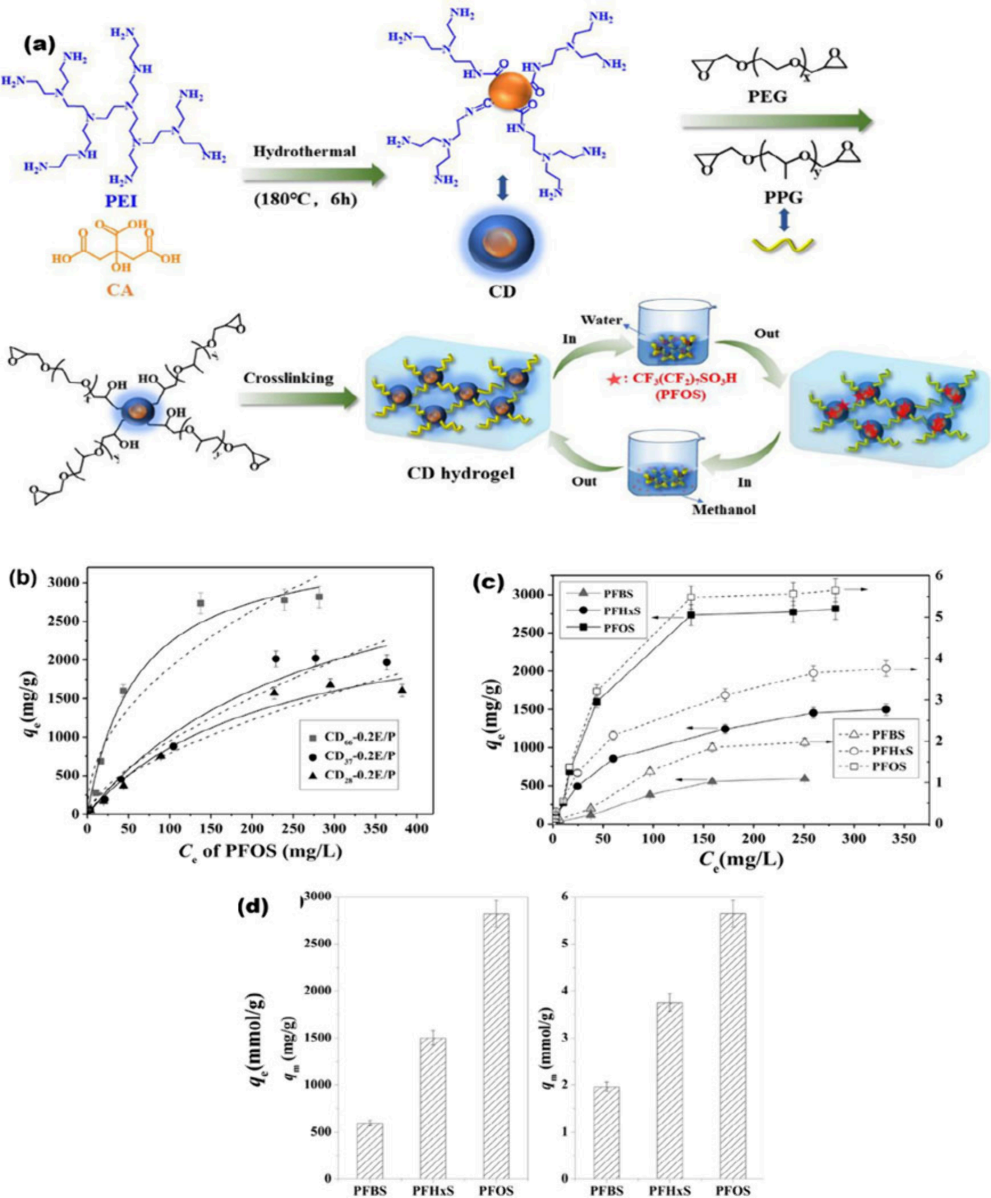
(a) Synthesis of CD-based hydrogels; (b) PFOS adsorption with varying CD contents (50 mg/L of adsorbent; pH 7.0; time: 48 h); (c) adsorption curves for PFOS, PFBS, and PFHxS for the CD_66_-0.2E/P hydrogel (50 mg/L of adsorbent; pH 7.0; 48 h); and (d) calculated maximum adsorption values from the corresponding adsorption isotherm curves.^109^ Reprinted with permission from ref 109. Copyright 2022 Elsevier.

### 3.2 Synthetic Polymer Hydrogels/Aerogels

Synthetic polymer-based hydrogels/aerogels are synthesized by using poly­(ethylene glycol) diacrylate (PEGDA), poly *N*-[3-(dimethylamino)­propyl]­acrylamide, poly­(*N*-isopropylacrylamide), 2-dimethylaminoethyl methacrylate, polyacrylamide, etc., synthetic polymers with a high water absorption and retention capacity without sacrificing structural integrity.^87,111^ These materials have been utilized in numerous applications like tissue engineering, stimuli-responsive materials, water treatment, and drug delivery because of their eco-friendly nature, a good response to environmental stimuli such as pH, temperature, or ionic strength and ability to mimic natural tissues.^112–115^

Huang et al.^115^ designed three different PEGDA-based reusable hydrogels to adsorb 2,3,3,3-tetrafluoro-2-(heptafluoropropoxy)­propanoic acid (GenX) and short- and long-chain perfluoroalkyl acids through amination, fluoridation, and/or bifunctionalization of PEGDA. Amination was carried out to increase the electrostatic forces between sorbents and PFAS by treating PEGDA with [2-(meth­acryloyl­oxy)­ethyl]-tri­meth­yl­ammo­nium chloride (MTAC). In contrast, fluoridation of PEGDA was carried out via treatment with 1*H*,1*H*,2*H*,2*H*-perfluorooctyl methacrylate (13FOMA) to enhance the hydrophobic interaction, and finally the bifunctionalization of PEGDA was carried out to enhance both the hydrophobic and electrostatic force interaction simultaneously by combining the approaches of amination and fluoridation techniques. Three different hydrogels, i.e., aminated (A-PEGDA), fluoridated (F-PEGDA), and aminated plus fluoridated PEGDA (AF-PEGDA), were tested for the adsorption of five different PFASs (PFOS, PFOA, PFBA, PFBS and GenX). The aminated PEGDA exhibited the maximum sorption capacity for three PFAS [PFOA: 109.9 ± 8.5 μmol/g; PFOS: 30.4 ± 0.4; PFBA: 199.5 ± 10.8; PFBS: 190.6 ± 21.0; and GenX: 86.7 ± 5.1 μmol/g], whereas bifunctionalized PEGDA showed the highest capacities for the remaining two, i.e., PFOA and GenX only [PFOA: 110.6 ± 9.7; GenX: X: 98.7 ± 3.9 μmol/g], confirming that both interaction forces contribute to the sorption. Fluorinated PEGDA sorbs very low levels of PFBA, PFOA, and PFOS in 6 h (<10%) and was unable to absorb GenX and PFBS; however, aminated PEGDA showed 100% adsorption against PFOA and PFBS and 78% and 91% sorption against PFBA and PFOS, respectively. The bifunctionalized PEGDA, compared to the aminated one, showed a lower sorption percentage. Furthermore, during the desorption study, it was noted that more than 90% of PFASS adhered to the surface of spent aminated and bifunctionalized PEGDA could be removed using 70% methanol containing 1% NaCl, confirming that PEGDA-based hydrogels can be regenerated for reuse.

Chaix et al., prepared three different 3D hydrogels, namely, a porous hydrogel without trianglamine (3D-SH) as the control [100% of P123 dimethacrylate (PDM)], a nonporous hydrogel trianglamine (3D-SHΔ; PDM + trianglamine), and a 3D porous hydrogel trianglamine (3D-PSHΔ; dimethacrylate-ureido-trianglamine mixture) by using the stereolithography technique. The 3D-PSHΔ aerogels exhibited a higher PFOA adsorption capacity (51.3 mg/g) compared to that of 3D-SH (33.55 mg/g). Further to enhance the 3D-PSHΔ removal efficiency, the free secondary amine of the Δ was quaternized to generate 3D-PSHΔQ+ by treating it with iodomethane. The 3D-PSHΔ and 3D-PSHΔQ+ hydrogels were tested against a series of (PFAS: PFOA, PFHpA, PFHxA, PFPeA, PFOS, and PFBS at an initial concentration of 0.0012 mmol/L) for 24 h, and the 3D-PSHΔQ+ hydrogel showed almost a hundred percent removal of all PFAS (PFOA: 99; PFHpA: 99; PFHxA: 98; PFPeA: 93; PFOS: 99; PFBS: 99%), better than its counterpart 3D-PSHΔ­(92, 95, 88, 83, 96, and 95%, respectively). Even when adsorption was carried out for 300 min, an impressive % removal was noted with 3D-PSHΔQ+ hydrogel for different PFAS (PFOA: 90%; PFOS: 95%; PFBS: 74%; PFHpA: 91%; PFHxA: 88%; and PFPeA: 89%) because of the higher affinity of PFOS for the positively charged surface of the quaternized hydrogel. Further, sorption isotherm data of both 3D-SHΔ and 3D-PSHΔ in 5 to 200 ppm in deionized water were noted to be best with the Freundlich isotherm, signifying a multilayer sorption and adsorption with different binding energies. 3D-PSHΔ followed pseudo-second-order kinetics in the case of deionized and river water at a PFAS concentration of 5 ppm; whereas 3D-SHΔ followed pseudo-first-order kinetics. These results confirm that diffusion of PFOA to the sorption sites, in the case of 3D-PSH, is not the primary rate-limiting step.

Ateia et al.^2^ prepared a poly­(*N*-[3-(dimethylamino)­propyl]­acrylamide methyl chloride quaternary (DMAPAA-Q) based hydrogel by mixing cationic monomer DMAPAA-Q (2.5 M), NNBAM (0.1 M), and the UV initiator, 2-oxoglutaric acid (0.05 M) together in deionized water for sequestering 16 PFAS from different classes from two surface waters (surface water raw and water treatment influent) and treated wastewater, with varying background anions (sulfate, nitrate and chloride ion) and dissolved matter composition, at adsorbent and adsorbate concentrations of 70 mg/L and <1000 ng/L, respectively (Figure 6a). The poly-DMAPAA-Q hydrogel exhibited a higher removal tendency, regardless of the PFAS chain length, toward sulfonated PFAS than carboxylic ones and fast removal kinetics, attaining equilibrium within 60–120 min (Figure 6b). These results were found to be consistent with density functional theory (DFT) results on the adsorption energy of long- and short- hain PFAS on poly-DMAPAA-Q hydrogel. DFT is a quantum-mechanical modeling technique that uses the electron density to ascertain the electronic structure (generally the ground state) of multielectron systems, such as molecules, atoms, and solids. For the calculation of adsorption energy, initially, they calculated the electrostatic potential of the hydrogel surface using a model pentamer of DMAPAA-Q, and it was found that the polymer is entirely positively charged, possessing higher positive regions near the quaternary ammonium groups compared to other regions of the polymer. Then, subsequently, the adsorption strength of five anionic PFAS adsorbates, namely Gen X, PFBA, PFOA, PFBS, and PFOS, was measured by positioning them over the highly positively charged quaternary ammonium group. The two sulfonic PFAS (PFBS, and PFOS) showed comparatively stronger exergonic energies (ranging from 79.95 to −82.59 kJ/mol) than three carboxylic PFAS (−11.65 to −28.27 kJ/mol). Furthermore, no impact of time on desorption was observed when the experimental time was increased to 24 h; removal efficiency was also unaffected by varying pH levels (pH ranged from 4 to 10). The poly-DMAPAA-Q hydrogel maintained good removal efficiency even after six consecutive adsorption/regeneration cycles (desorption was carried out in 50 mL NaCl/methanol solution).

**Figure 6:**
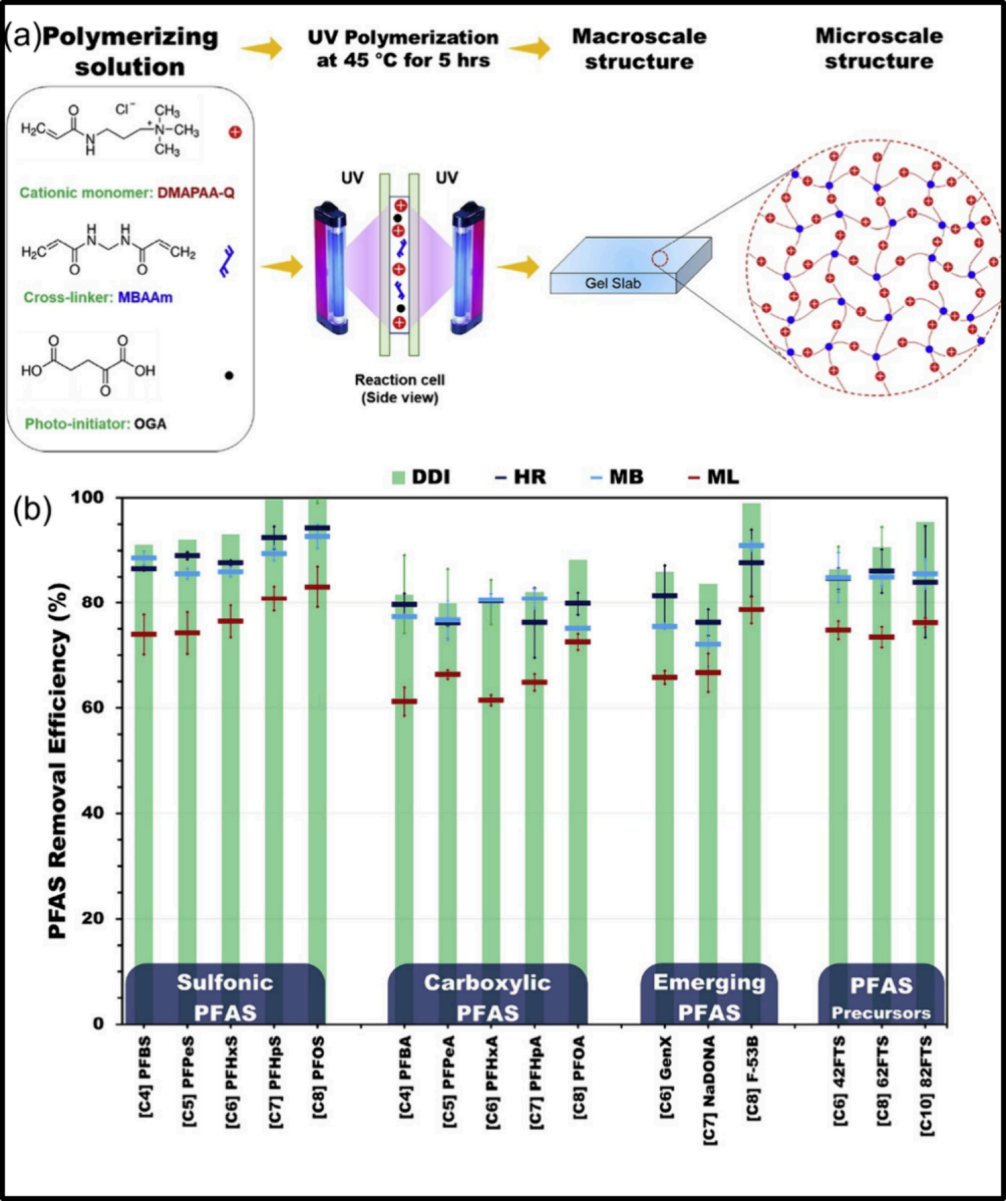
(a) Synthesis of poly DMAPAA-Q hydrogels and (b) adsorption ability of synthesized hydrogels (70 mg/L) against 16 PFAS (1000 ng/L) in distilled deionized water (DDI), lack water (HR), influent to WTP (MB), and treated wastewater (ML). DOC = 2.4 ± 0.3 mg/L, pH 6.5, and equilibrium time of 24 h.^2^ Reprinted with permission from ref 2. Copyright 2019 Elsevier.

Aminated polyacrylamide hydrogel foams, namely PAM–PANI-3, PAM–PANI-2, and PAM–PANI-1, were developed by initially cross-linking polyacrylamide (PAM) and polyaniline (PAN) and subsequently pyrolyzing the obtained PAM–PAN hydrogels at varying temperatures of 413, 301, and 133 °C for 2 h, respectively.^116^ The idea of inclusion of AN was to introduce positive charges and thus the electrostatic interaction between PFAS and the adsorbent. Among all samples, PAM–PANI-2 (0.04166 mg/mg) gives the best adsorption capacity, followed by PAM–PANI-1, PAM–PANI0 (no pyrolysis), PAM (0.01330 mg/mg), and PAM–PANI-3 (0.00734 mg/mg). Further, PAM–PANI-2 showed a remarkable regeneration ability of 92.3% for PFOA even after a five-cycle sequential experiment, using a MeOH and NaCl mixture as a regeneration agent. Based on the adsorption isotherm experiment (the kinetics study confirmed pseudo-second-order adsorption), it has been reported that all adsorbents followed the Langmuir adsorption model, indicating monolayer adsorption of PFOA on their surface. When used for the removal of seven different types of PFAS, PAM–PANI-2 was able to adsorb greater than 90% of each PFHxA, PFHpA, PFOS, and HFPO-TA, while a removal percentage of 82% for PFBA, 31% for TFA, and 51% for PFdiCA was noted after 1 h of the experiment.

### 3.3 Ionic Fluorogels

Ionic fluorogels, a new class of polymeric adsorbents, leverage synergistic fluorous and electrostatic interactions to achieve high capacity, strong affinity, and rapid uptake of diverse PFAS from real waters. They have emerged as promising candidates for remediating both legacy and emerging PFAS. Their synthesis shows that end-group substitution can readily tune hydrolytic stability, while network architecture modifications significantly influence the decontamination performance in simulated natural waters.

Kumarasamy et al.^117^ developed ionic fluorogels (IF-X; X = wt % of amine containing group)—hydrogels combining fluorinated segments and ionic groups—via radical polymerization of perfluoropolyethers (PFPE; fluorophilic matrix) with 2-dimethylaminoethyl methacrylate (DMAEMA, 10–60 wt %; amine containing monomer), using Fluorolink MD 700 as a cross-linker and azobis­(isobutyronitrile) (AIBN) as an initiator to remove 21 different PFAS. To enhance the ion-exchange capacity, tertiary amine IFs were further quaternized with methyl iodide, yielding permanently charged variants (IF-X^+^; X+ = wt % of ammonium comonomer incorporated). For comparison, nonfluorous ionic gels (INF) were prepared from DMAEMA and polyethylene glycol dimethacrylate (PEGMA).

In simulated natural water (NaCl 200 mg/L, humic acid 20 mg/L), quaternary and tertiary IFs (used as 100 mg/L of adsorbent, or IFs for 21 h) removed >80% of PFAS (water spiked with short chain PFHxA, three long chain PFOA, branched GenX at 50 μg/L of each adsorbate), outperforming INFs. At environmentally relevant concentrations (1 μg/L PFAS, 10 mg/L IF, 21 h), IF-X^+^ (20–40 wt %) achieved >80% removal of short-chain PFAS (PFHxA) and GenX. The best-performing IF-20^+^ exhibited rapid GenX uptake: 86% in 30 s, 90% in 30 min at 200 μg/L adsorbate concentration (adsorbent dosage:100 mg/L), and 94% in 30 min at 1 μg/L (adsorbent dosage: 10 mg/L), with no desorption after 72 h. Adsorption of GenX followed the Langmuir isotherm and pseudo-second-order kinetics, and IF-20^+^ retained high activity over six regeneration cycles.

In real water matrices (20–50 ng/L PFAS), IF-30^+^ (dosage: 100 mg/L) showed >95% removal of sulfonic acids (PFBS, PFHxS, PFOS), indicating preferential affinity for sulfonates, while removal of short-chain carboxylates (PFPeA, PFBA) was lower (87% and 60%, respectively).

In a follow-up study, the group enhanced IF stability via copolymerization of fluorinated comonomers [pentafluorostyrene (PFS)-Fluorolink E10H, PFS-fluorinated tetraethylene glycol (FTEG), and an amine (2-(dimethylamino)­ethanol)-substituted PFS derivative], followed by quaternization.^118^ Among different IFs (IF-X; X = 1 to 10, used for variable wt % composition of three comonomers, PFS-E10H, PFSFTEG, and amine substituted PFS), IF-8 (30:30:30 composition) demonstrated superior removal of short-chain PFAS (conventional settled water spiked with allowed levels of PFHxA, PFOA, and GenX of concentration 500 ng/L each) in mini-rapid small-scale column tests (RSSCTs), outperforming commercial ion-exchange resins. In batch tests, IF-1 (80:0:20 wt % composition; 100 mg/L) removed 77% of the 18 PFAS out of total 21 PFAS spiked at 1 μg/L each, with near-complete removal (>98%) of long-chain PFAS (≥C7), moderate removal (87 to 97%) of four perfluorocarbons (PFBS, PFPeA, PFO3OA, PEPA, NVHOS), and lower selectivity for ultrashort PFAS (C2–C3; PMPA, PFBA, PFO2HxA, and PFMOAA). Adsorption data again fit the Langmuir model, and recyclability exceeded 75% after five cycles.

### 3.4 Biopolymer–Polymer Hybrid Hydrogels/Aerogels

Numerous polysaccharides, such as sodium alginate (SA), CS, cellulose, etc., have been extensively used for the development of hydrogels/aerogels.^119^ These hydrogels/aerogels possess several advantages over the synthetic ones, such as eco-friendly nature and good biodegradability and biocompatibility, making them an attractive choice for a variety of applications.^82,85,120,121^ Ilango et al.^122^ prepared 7 different types of polyethylenimine (PEI) modified sodium alginate (ALG) and CS-based aerogels of variable composition using epichlorohydrin and glutaraldehyde (GTH) as cross-linkers, namely ECH–CSPEI-1 (wt % ratio of CS:PEI:ECH = 47.18:37.75:15.07), ECH–CSPEI-2 (CS:PEI:ECH = 64.11:25.65:10.24), ALGPEI-1 (ALG:PEI:GTH = 20:50:3), ALGPEI-2 (ALG:PEI:GTH = 13.33:66.67:20), ALGPEI-3 (ALG:PEI:GTH = 8:80:12), GTH–CSPEI (CS:PEI:GTH = 8:80:12), and CTAC-ALGPEI (ALG:PEI:CTAC:GTH = 7.94:79.37:11.90:0.79). When used for the adsorption (adsorbent dosage: 100 mg/L) of a mixture of 12 different PFASs in water (9 long- and short-chain PFAAs, GenX and 2 precursors) at PFAS concentrations ranging from 20 to 500 μg/L in the case of the adsorption isotherm study and 10 μg/L in the case of the adsorption experiment, for each, GTH CSPEI and ALGPEI-3 aerogels outperformed others at the wide range of pH 2 to 10 and showed remarkable adsorption capacities of 12,133 and 3045 mg/g, respectively (calculated using the Sips model). After detailed characterization and analysis of the adsorbents before and after PFAS adsorption, the hydrophobic interaction was reported to be dominant over the electrostatic interaction (playing a minor role only) during PFAS sorption. Compared to other aerogels, a nonsignificant impact of time on ALGPEI-3 aerogel equilibrium adsorption ability was noted; in only the first hour, this aerogel was able to remove cent percent of perfluoroundecanoic acid (PFUnA), PFNA, PFOS, perfluorodecanoic acid (PFDA), and *N*-ethyl perfluorooctane sulfonamido acetic acid (2-N-EtFOSAA), the best among all the sorbents against these five PFASs. Its tendency to remove the PFOA, PFHxS, and 6:2 FTSA was close to that of GTH–CSPEI, while for removal of short chains such as PFHpA, PFHxA, PFBS, and GenX, GTH–CSPEI was on edge compared to ALGPEI-3. Further, GTH–CSPEI also outperformed all of the aerogels in the removal of PFHxA, PFHpA, PFBS, PFOA, 6:2 FTSA, PFHxS, and GenX. Additionally, for both GTH CSPEI and ALGPEI-3, the Freundlich model was found to be the best fit across all ranges of PFAS concentrations.

The same group in another study converted chunks of ECH–CSPEI aerogels (average size: 13.4 mm) into flakes of an average size of 9 mm by cutting and subsequent grinding, thereby enhancing the tendency of the parent aerogels to remove short-chain PFAS and GenX.^123^ The obtained flakes, when immersed in an amount of 25 g (500 mg/L) in 50 mL PFAS solution comprising all 12 PFAS [PFSAs (C4, C6, and C8), PFCAs (C6–C11), GenX, 6:2 FTS, and 2-(*N*-ethylperfluorooctanesulfonamido)­acetic acid (2-NEtFOSAA)], exhibited >99% removal efficiency against all short- and long-chain PFAS and >97% for GenX after 10 h of immersion. Furthermore, the flakes retained a good removal efficiency even after four consecutive cycles of regeneration (regenerated by extraction with 2% methanolic ammonium hydroxide) and use against all PFAS (Figure 7a). When immersed in PFAS-spiked tap water (spiked at initial concentrations of 30, 70, or 100 ng/L each), the flakes removed long-chain PFAS almost entirely in 1 h; however, the short-chain PFAS, including PFHxA, PFHpA, PFBS, and GenX, required a comparatively longer time (Figure 7b). The flakes were able to remove >95% of these tough-to-remove PFAS in 24 h, with the exception of GenX, at an initial 100 ng/L PFAS concentration, even after 48 h. Further, through DFT analysis, strong stabilizing electrostatic and hydrophobic interactions between the adsorbate and adsorbent were confirmed, with binding energies ranging between −41.3 to −48.5 kcal/mol for PFOS, −24.0 to −30.1 kcal/mol for PFOA, and −40.5 to −47.3 kcal/mol for PFBS.

**Figure 7:**
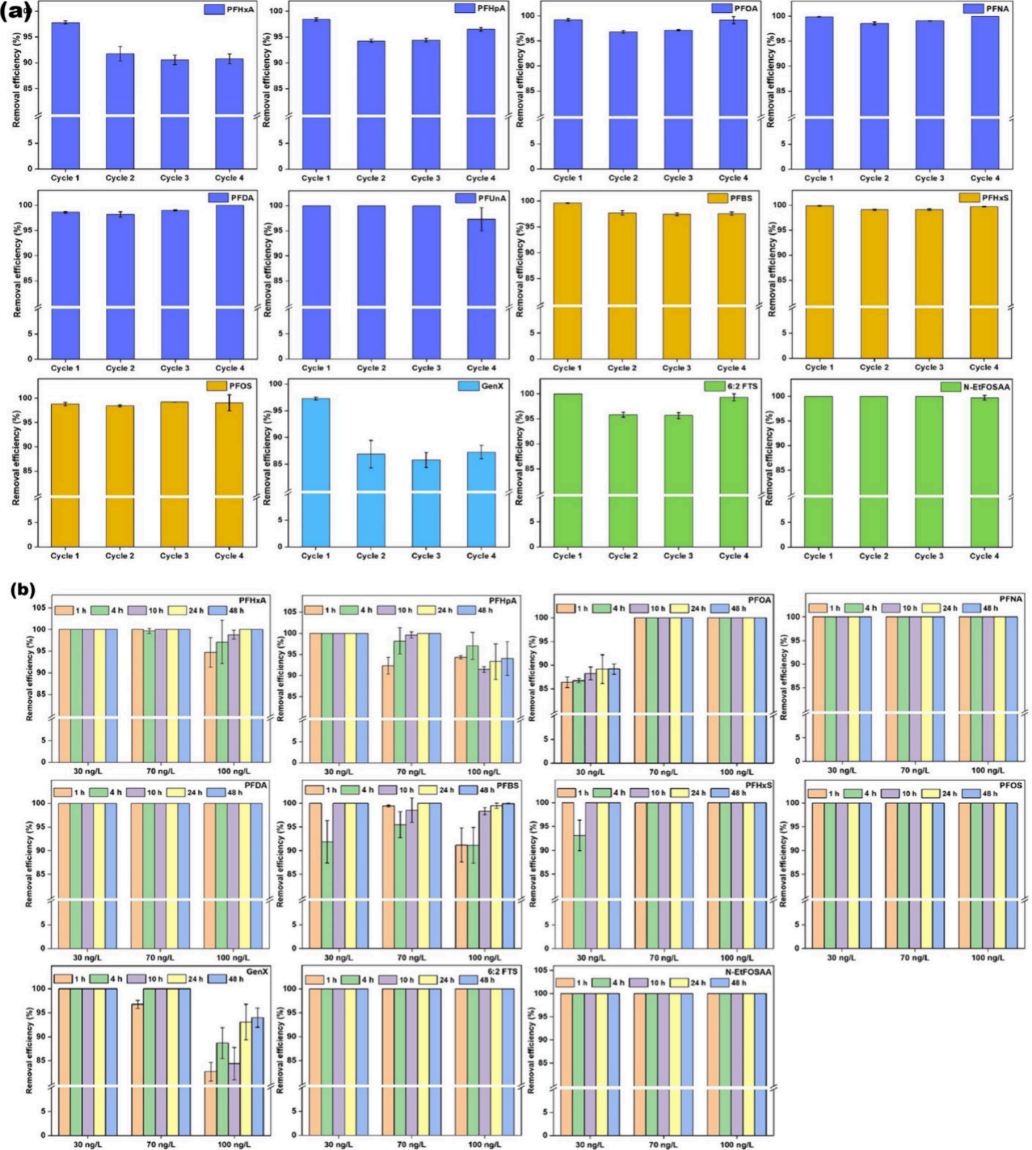
Removal efficiency of ECH–CSPEI flakes against (a) PFAS mixtures after four cycles of regeneration and reuse [initial PFAS concentration: 10 μg/L; adsorbent dosage: 500 mg/L][Bibr ref123] and tap water spiked with PFAS [initial PFAS concentration: 30, 70, or 100 ng/L; adsorbent dosage: 500 mg/L].^123^ Reprinted with permission from ref 123. Copyright 2024 Elsevier.

Alaska et al.,^124^ prepared two types of eco-friendly and cost-effective gravity-derived κ-carrageenan (kC)-based hydrogel membranes, i.e., 3 wt % kC 0.3 wt % AC (kC:AC) and 3 wt % kC:3 wt % vanillin (kC:V) of different thicknesses of 0.5 to 2 cm for removal of PFOA from actual and synthetic wastewater using a gravity column. kC-AC hydrogel showed better rejection ability (86.9% at pH 4) than the kC-V hydrogels (85.7% at pH 4) against a PFOA feed solution with a concentration of 1.5 mg/L. Further, when 2 cm thick hydrogels of each adsorbent were used for soil remediation wastewater treatment (containing 179 mg/L PFOA), the kC-AC hydrogel removed approximately 81.1% of PFOA, while the kC-V hydrogel removed 79.3% of PFOA at a water flux of 25.6 and 21.5 LMH, respectively. Recently, Wang et al.,^125^ developed a NF membrane via designing a hydrogel layer with cross-linking of CS with glutaraldehyde in the first step and subsequently grafting polyamine (PA) on its surface through an interfacial polymerization technique to customize NF membranes with enhanced PFAS rejection ability (achieved a maximum of 83–89% and 90% rejection for short-chain and long-chain PFAS, respectively). The significant difference in long- and short-chain PFAS removal by this membrane has been attributed to various factors, including size, electrostatic attraction/repulsion, sieving effect, and hydrophobic attraction, among which the size sieving effect generally dominates when the PFAS molecular weight exceeds the pore size of the membrane. Further, membranes facilitate better passage of mineral ions because of the strength of electrostatic attraction; however, a 50% decrement in passage of mineral ions was noted compared to the control one.

CTAB-alginate hydrogels, fabricated by Shaikh and Nawaz,^126^ exhibited a removal efficiency of 94.8% for PFOA when dipped in a 50 mg/L concentrated PFOA solution. The experimental data were found to be closely aligned with the Sips isotherm model (a combined model of Langmuir and Freundlich adsorption isotherms, also known as the Langmuir–Freundlich isotherm) and the pseudo-second-order kinetic model, indicating a maximum possible adsorption capacity of 382.1 mg/g. Such a high adsorption has been attributed to hydrophobic and electrostatic interactions and hydrogen bonding. The Sips model signifies that at low adsorbate concentrations hydrogels follow the Freundlich adsorption isotherm and at higher concentrations they follow the Langmuir adsorption isotherm. Furthermore, hydrogels demonstrated high selectivity toward PFOA and, upon treatment with a regenerant solution of ethanol/ammonium hydroxide, were able to regenerate 80% of their adsorption capability, which declined to 70% after three consecutive adsorption–desorption cycles. The hydrogels removed approximately 80% of PFOA is selectively removed from river water at a concentration of 500 μg/L PFOA.

SA and β-cyclodextrin-based hydrogels (SA-β-CDEX) have been utilized to remove PFOS by Zakaria et al.^127^ A 1280 mg amount of SA-β-CDEX hydrogels was administered under ideal conditions, including a contact time of 30 min, a pH of 5.5, a temperature of 70 °C, and a 10.0 ppm PFOS solution. The maximum removal efficiency of 84.72% and adsorption capacity of 0.0764 mg/g for PFOS have been achieved using these hydrogel beads. The experimental data were reported to be best fit with the Langmuir isotherm model and the pseudo-second-order kinetic model. In a follow-up investigation, the same group added carbon nanotubes (CNT) to SA-β-CDEX hydrogels and reported an increase in PFOS removal tendency to 91.6% for the resulting hydrogels (SA-β-CDEX/CNT), as evaluated via a batch adsorption experiment.^128^ The optimum parameters for PFOS removal were reported as follows: adsorbent dosage: 1000 mg; 10 mg/L of PFOS solution; contact time: 45 min; pH 3. Kinetic and adsorption isotherm data were found to perfectly fit the same models as those in the case of SA-β-CDEX hydrogels.

Shahrokhi and Park^129^ used two types of CS-based hydrogels, i.e., epichlorohydrin cross-linked CS beads and PEI-grafted-CS beads (GCB_S_), after crushing for the adsorption of two long- and short-chain PFAS_S_, namely PFOA and PFOS, and PFBA and PFBS, respectively. Out of two hydrogels, the latter one showed superior performance against all the PFAS during the batch adsorption experiment in the aqueous phase, showing maximum Langmuir adsorption capacities of 500, 555.5, 769.2, and 1428.6 μmol/g for PFOS, PFOA, PFBS and PFBA, respectively. However, during the actual water batch adsorption experiment, the adsorption ability of GCBS was observed to be drastically reduced under real water conditions. Further, regenerated GCBs, for which regeneration was done via treatment with a solution of ethanol and NH_4_OH, showed a stable sorption for PFOS and PFOA in four cycles, whereas following the first regeneration their sorption capacity toward the short-chain PFBA and PFBS declined sharply by 74.6% and 43.9%, respectively; however, in the following three cycles, it was noted to remain relatively steady.

### 3.5 Hybrid and Synthetic Thermosensitive Hydrogels

Thermosensitive hydrogels are a new kind of novel adsorbents, in which adsorbed contaminants can be desorbed by changing environmental conditions without treating them with chemical reagents. Wang et al.^130^ prepared three different types of thermosensitive hydrogels for the possible removal of PFOS, namely poly­(*N*-isopropylacrylamide) (PNIPAM)/poly­(methyl methacrylate) (PMMA)/CS-1, 2, and 3 (X = 1, 2, and 3 correspond to 2, 3, and 4 wt % concentrations of CS, respectively) hydrogels, using *N*-isopropylacrylamide (NIPAM; 1 g), methyl methacrylate (MMA; 0.1 g) and varying concentrations of CS i.e., 2, 3, and 4 g solutions, respectively, through one pot reaction. Among the various developed hydrogels, PNIPAM/PMMA/CS-3 exhibited the best performance, achieving a maximum absorbance of 476.5 mg of PFOS/g at pH 4. This highest removal has been attributed to an enhancement in hydrogen bonding and electrostatic interactions between hydrogels and PFOS, accompanied by an increase in the CS amount. At pH 4, −NH_2_ groups lying on the CS surface (maximum in case of PNIPAM/PMMA/CS-3) undergo protonation to −NH_3_^+^ species, thus enhancing their ability to electrostatically attract with −SO_3_^–^ groups of PFOS. Further, it has been confirmed that adsorption isotherm follows the Langmuir adsorption model. Desorption of PFOS was carried out in distilled water at 323 K, and after five consecutive adsorption–desorption cycles, the PNIPAM/PMMA/CS-3 hydrogel was able to retain 95% of its original adsorption ability.

Saad et al.^131^ also fabricated thermosensitive pure PNIPAM hydrogels via radical-initiated polymerization of the NIPAm monomer using ammonium persulfate as an initiator and NNMBA as cross-linkers and subsequently used them as it is or after filling the PVDF membrane for the removal of PFOA. The PNIPAm is widely utilized because of its phase transition ability from a hydrophilic hydrated to a dehydrated state over its lower critical solution temperature (LCST) of 32 °C (Figure 8a). Below the LCST, PNIPAM hydrogels were noted to undergo swelling in an aqueous environment (hydrogen bonding interactions); however, as the temperature is increased beyond the LCST value, the isopropyl groups are replaced by backbone dehydrates, leading to an increased interaction between PFOA and the adsorbent due to hydrogen bonding and dispersion interaction. Above 35 °C, the Freundlich distribution coefficient (*K*_d_) was reported to be 0.073 L/g, whereas at a lower temperature (22 °C) its value was found to be 0.026 L/g. Further, kinetic analysis revealed that the adsorption fit pseudo-second-order kinetics quite well, yielding second-order rate constants (*k*_2_) of 0.012 and 12.6 g/mg/h for adsorption and desorption, respectively. The PNIPAM-functionalized PVDF membrane exhibited a steady adsorption (at 35 °C) and desorption capacity (at 20 °C) over 5 consecutive cycles (desorption was conducted by treating with deionized water at 20 °C). A 60% PFOA desorption percentage was achieved in pure water below the LCST during the first cycle; however, in subsequent cycles, > 90% of adsorbed PFOA was desorbed. Frazar et al.^132^ found an increase in the LCST value for the PNIPAM hydrogel after surface modification using different cationic monomers (3-acrylamidopropyl)­trimethylammonium chloride (DMAPAQ) and *N*-[3-(dimethylamino)­propyl]­acrylamide (DMAPA). Their idea was to equip the hydrogel with positively charged sites for better interaction and bonding with deprotonated PFOA molecules through electrostatic interactions (Figure 8b). An increase in % removal of PFOA from 86.9 to 95.9% and 94.9% after modification using DMAPA and DMAPAQ, respectively, was noted at pH 4.

**Figure 8:**
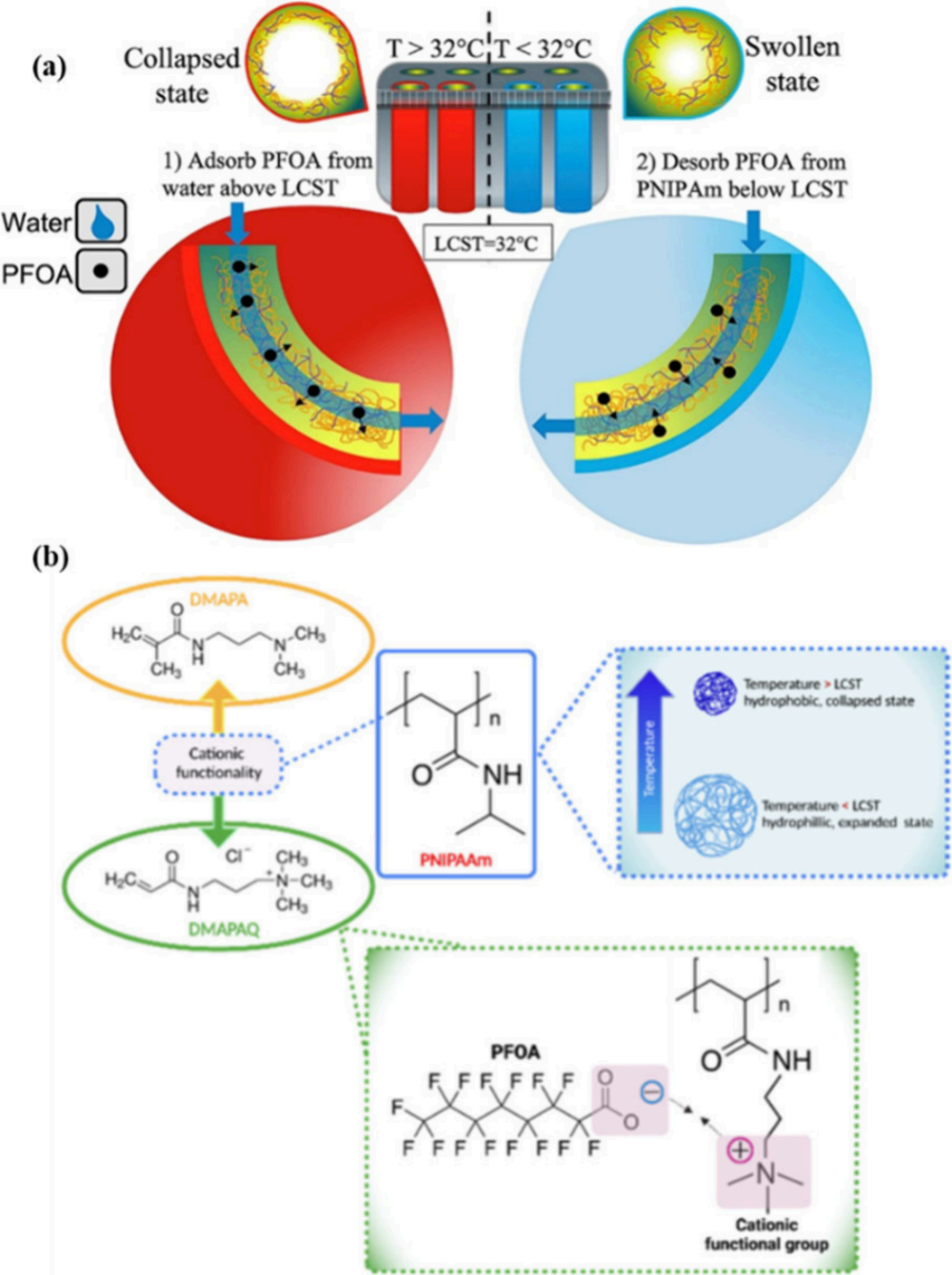
(a) Change in adsorption behavior of PNIPAM hydrogels for PFOA above and below the LCST.^131^ “Reprinted with permission from ref^131^. Copyright 2020 Elsevier. (b) Cationic surface modification of the PNIPAM hydrogel with a cationic monomer for better bonding with PFAS molecules.^132^ Reprinted with permission under CC BY-4.0 from ref 132. Copyright 2022 MDPI.

Among the different types of hydrogels or aerogels, hybrid hydrogels, particularly those made up using biopolymers (CS, alginate, and carrageenan), have many advantages over synthetic ones, such as being less costly, biocompatible, and biodegradable. Owing to their porous interconnected structure, these hydrogels have shown improved diffusion and efficient adsorption of PFAS molecules. Additionally, CS-based hydrogels,^122^ due to the presence of amine groups, were found to remove PFAS more effectively than other hydrogels, occupying second place among all hydrogel-based adsorbents. CD_66_–0.2 PEG/PPG hydrogels,^109^ developed in 2022 for PFOA removal, are still the top performers, even compared to commercially available PAC (adsorption capacity 520 mg/g for PFOA),^133^ granular AC (390 mg/g),^134^ and single-walled carbon nanotubes (712 mg/g)^135^ for PFOS.

## 4 Adsorption Kinetics and Adsorption Isotherm

Researchers have applied various empirical isotherm models, including Freundlich, Langmuir, Sips, and statistical physics models, to elucidate PFAS adsorption behavior on hydrogels. Unlike thermodynamic models (e.g., BET), which are grounded in fundamental material properties, these empirical models rely on experimental data to describe the adsorption capacity, mechanism, and adsorbent–adsorbate interactions. When the Langmuir model fits well, a homogeneous surface with finite active sites and monolayer adsorption is indicated, while alignment with the Freundlich model suggests heterogeneous surfaces and multilayer adsorption. The Sips model, a hybrid of Langmuir and Freundlich, captures both scenarios: at low PFAS concentrations, it reflects Freundlich-type multilayer adsorption, while at higher concentrations it converges to Langmuir-type monolayer adsorption.

Hydrogels or aerogels, including AGO gels,^107^ 3D-SHΔ,^71^ 3D-PSHΔ,^71^ and PNIPAM,^131^ were noted to follow the Freundlich adsorption isotherm; PAM–PANI-2,^116^ IF-20+,^117^ IF-1,^118^ SA-β-CDEX,^127^ GCBs,^129^ PNIPAM/PMMA/CS-3,^130^ and SA-β-CDEX/CNT^128^ obeyed the Langmuir adsorption isotherm; and the CTAB-functionalized alginate hydrogel^126^ followed the Sips adsorption isotherm. Through the kinetic study of adsorption, one can find the rate-determining step and thus may assign a possible mechanism of adsorption. The best fit of experimental data with first- and second-order kinetics confirms the physical and chemical adsorption, respectively. Considering the findings of multiple researchers, it can be concluded that except for 3D-SHΔ76, the majority of hydrogels/aerogels follow the chemical adsorption pathway.

## 5 Impact of Temperature and pH of the Reaction

There are numerous factors, including adsorbent dosage, PFAS concentration, temperature, time, and solution pH, that impact PFAS adsorption. However, in the present article, two of the most critical factors, temperature and pH, have been thoroughly reviewed. A couple of researchers reported contrary results regarding the impact of the temperature on the adsorption of PFAS by hydrogels. Liu et al. reported a decrease in the density (from 25.51 to 12.54 mg/g) of adsorption layers of PFOA and the number of receptor sites on Cu/F-rGA with an increase in temperature from 20 to 40 °C.^100^ Similarly, Tian et al.^107^ also found a decrease in adsorption capacity from 1575 to 577.1 mg/g with increasing temperature (298 to 318 K, respectively), indicating exothermic adsorption of PFOA onto AGO aerogels. Long et al.,^110^ contrary to above finding reported an increase in PFOA adsorption on CEGH hydrogels with an enhancement in temperature from 20 to 40 °C, attributed to the activation of active sites at a higher temperature. When the temperature was approximately 40 °C, approximately 1158 mg/g adsorption capacity was noted.

The pH of the solution alters the chemical properties of the adsorbate as well as the adsorbent, which in turn impacts the adsorption properties of the adsorbent. Numerous researchers have evaluated the impact of solution pH on the performance of the hydrogels. Illango et al.^122^ found that the pH value between 2 to 10 of the solution did not affect the adsorption ability of ALGPEI against four PFAS, i.e., PFOS, PFNA, PFDA, and 2-N-EtFOSAA. However, a lower pH (1.0) tends to decrease the adsorption. Similarity for PFUnA, no impact on removal efficiency was noted in the pH range of 4 to 10; however, a decrease was noted at pH 1 and 2. For other hydrophilic PFASs, namely, PFBS, PFHxS, GenX, and 6:2 FTSA, the highest removal efficiency was observed at a pH of 4.0. The Cu/F-rGA hydrogels demonstrated electropositivity in solution with a pH below the zero potential point (pHpzc: 6.3), which means they preferentially attract PFOA anions due to electrostatic attraction. At higher solution equilibrium pH, or higher than pHpzc, the Cu/F-rGA surface becomes negatively charged and thus inhibits the PFOA adsorption due to electrostatic repulsion between the PFOA anions and the negatively charged adsorbent surface.^100^ Tian et al.^107^ discovered a reduction in PFOA removal effectiveness with AGO aerogels from 99.9% to 89.4% with an increase in pH from 1.60 to 9.26. This behavior has been attributed to enhanced electrostatic repulsion between the AGO aerogel and PFOA at higher pH because of the generation of the electronegative surface on the AGO aerogel. Wang et al.^109^ reported a decrement in the adsorption ability of CD_66_–0.2PEG/PPG at pH higher than the pHpzc (noted at pH higher than 9) for PFOS.

Alsaka et al.^124^ discovered a decrease in rejection of PFOS with a kC-V hydrogel-based membrane from 85.7% to 79.5% with an increased feed solution pH from 4 to 10. Shaikh and Nawaz^126^ reported the point of zero charge (pHzpc) for the CTAB-alginate hydrogel at pH 7. They concluded that the ability of the CTAB-alginate hydrogel to remove PFOA was not significantly impacted by pH values ranging from 2 to 9. The phenomenon has been attributed to the presence of positively charged quaternary ammonium groups in CTAB, which enable the capture of anionic PFOA molecules across this broad pH range, ensuring adsorption even under alkaline pH conditions. However, the adsorbent’s capacity to remove PFOA was found to significantly decline at pH > 9. Long et al.,^110^ reported a continuous decrease in adsorption capacity from 1024 mg/g to zero upon an increase in pH from 2 to 10. The highest adsorption at pH 2 has been assigned to increased ionic interaction between the protonated amine in hydrogels and carbonyl groups of PFOA. Shahrokhi and Park,^129^ during their study on GCBs hydrogels, also reported a high adsorption of PFAS in acidic conditions (at pH 5 and 6) because of increased interaction in between the protonated amine group and deprotonated PFAS. On further decreasing the pH, the adsorption was noted to decrease, attributed to incomplete deprotonation of PFAS (optimum deprotonation was noted at pH 6). A maximum removal of 92%, 99%, 31%, and 75% was noted for PFOA, PFOS, PFBA, and PFBS, respectively, at pH 6.0, and was found to reduce to 40.3%, 43.8%, 81.6%, and 93% upon an increase in pH to 11.

## 6 Challenges

Despite promising laboratory-scale results, three major hurdles limit the translation of hydrogel-based PFAS adsorbents to real-world applications: adsorption selectivity, regeneration of spent hydrogels, and scalability.

Most studies report recyclability up to 5–6 cycles, with rare cases reaching 10 cycles. Solvent-assisted methods (acetonitrile, ethanol, methanol) and inorganic regenerants (NaOH, NH_4_Cl, NH_4_OH, NaCl, KCl) are typically used, either alone or in combination.^115,126^ For instance, Shaikh and Nawaz achieved a maximum of ∼81% regeneration in the first cycle (out of different regenerants ethanol + NH_4_Cl, ethanol + NH_4_OH, ethanol + NaOH, ethanol, NaOH, NH_4_OH, and NaOH) using ethanol + NH_4_OH for PFOA-loaded CTAB-functionalized alginate hydrogels, but efficiency dropped to ∼70% by the fourth cycle. While some hydrogel systems retain >90% removal after 5 successive recycles.^116,123,131^ Large-scale regeneration without a loss of structure or function remains unresolved. Conventional strategies—solvent washing, pH adjustment, or temperature control, may not always be effective at large scale.

Natural organic matters (NOMs) such as oxalic acid, formic acid, fulvic acid and humic acid, as well as carbonate, phosphate, and silicate found in surface water, can interfere with PFAS adsorption by competing with them for the available adsorbent sites on the hydrogel’s surface, even when the hydrogel is negatively charged.^73,126^ The poor adsorption of PFAS onto the hydrogel’s surface may be due to a lesser hydrophobic character and a fewer number of positively charged adsorption sites onto the adsorbent surface.^73^ The adsorption of NOMs onto hydrogel’s surface reduces the adsorption tendency of hydrogels due to electric repulsion between similarly charged adsorbed NOMs and adsorbate (PFAS). Furthermore, the decrement in the hydrophobic character of hydrogels may be due to the blockage of the maximum number of hydrophobic adsorbing sites by these NOMs (fulvic acid and humic acid). Ateia et al.^2^ reported 5–10 wt % decrement in PFOA removal on doubling the concentration of dissolved organic matter. Similarly, in the presence of oxalic acid and formic acid, the removal efficiency of PAM–PANI toward PFOA was noted to decline by 123%.

The presence of different inorganic salts is another factor, whose concentrations in various waters vary from region to region as well as from source to source. Most salts have adverse impacts on PFAS adsorption because of the double-layer compression effect (screening effect), which may decrease the strength of interactions in some cases between PFAS and positively charged hydrogels. Anion and cation complexation must be fully considered, as divalent cations show a greater inhibition impact than monovalent ions on PFAS adsorption. Ateia et al.,^2^ reported a marginal decrease in Poly DMAPAA-Q hydrogels adsorption capability for long-chain PFAS with an increase in background anions (NO_3_^–^,Cl^–^, and SO_4_^2–^) concentrations. However, for short-chain PFAS, more specifically carboxylic PFAS, the opposite trend was noted. Their finding confirmed the domination of hydrophobic interaction in the case of long-chain PFAS, and electrostatic interaction in the case of short-chain PFAS adsorption. Xu et al.^116^ noted a decrement in the PAM–PANI hydrogel’s ability to adsorb PFOA from 97.5% to 92.1%/86.3% and to 68.4%/55.2% with the addition of 5 mmol/L and 10 mmol/L each of CaCl_2_ and NaCl, respectively. The better performance of Ca^2+^ ions at the same concentration of Na^+^ ions may be due to the cation bridging effect of the divalent cation with PFOA’s carboxyl group.^136,137^ The CD_66_-0.2E/P hydrogel, when used in amount of 25 mg/L for the treatment of the simulated water containing a low concentration of PFOS (518.4 ng/L), NaCl (200 mg/L), and humic acid (20 mg/L) was able to remove approximately 86.6% of PFOS (less than the control one) within half an hour, and thereafter it remained almost constant.^109^ From the above discussion, it can be concluded that salts (anions) not only compete with PFAS but also impact adsorption through electrical double-layer compression (screening effect).

Despite extensive lab studies on hydrogel adsorbents for PFAS, most tests use unrealistic conditions. Future research should focus on realistic matrices, not at extreme concentrations of hydrogels, pH, PFAS concentration, and background chemicals such as organic materials, salt ions, and anions. The efficiency and selectivity of hydrogels toward specific target PFAS molecules can be enhanced selectivity via structural and surface modifications. Researchers should find new ways to lessen the cost of precursors and procedures used to produce hydrogels to make them more economically competitive than traditional PFAS removal technologies, such as biomass-based adsorbents, AC, membranes, or ion-exchange resins.

There is no doubt that numerous hydrogel-based adsorbents have shown excellent performance in the removal of PFAS, such as CD_66_–0.2 PEG/PPG,^109^ CEGH,^110^ and GTH–CSPEI aerogel;^122^ however, their synthesis involves an energy-consuming process (freeze-drying). ECH–CSPEI aerogels have shown remarkable potential in removing both long- and short-chain PFAS; however, for regeneration, a combination of organic solvents and salts is required, which adds additional cost to the technology. Therefore, techno-economic assessment would be useful in determining the economic feasibility of specific hydrogels/aerogels-based adsorbents utilized in the PFAS removal process. A lifecycle cost analysis (LCA) should also be conducted to understand the total costs associated with the synthesis, application in the real world, maintenance, transportation, regeneration, and disposal of hydrogels. It is a useful tool for quantifying and assessing the environmental effects of synthetic or hybrid hydrogels. It is important to assess how the entire hydrogel manufacturing and consumption process affects the environment, considering energy utilization and CO_2_ emissions, before scaling up.

## 7 Conclusion

This Review presents the synthesis, adsorption behaviors, mechanisms, challenges, and future prospects of hydrogels, aerogels, and foams for PFAS removal. Adsorption depends not only on adsorbent properties (surface area, pore volume, hydrophobicity, and functional groups) but also on solution chemistry, including pH, temperature, and background impurities. Enhanced electrostatic interactions at lower pH improve adsorption, whereas competing ions and organic matter can reduce the capacity.

While conventional adsorbents such as clays, cellulose, carbon nanotubes, graphene, activated carbons, and ion-exchange resins have been widely applied, hydrogels, and aerogels offer advantages, including high surface area, tunable surface chemistry, improved selectivity, regeneration, and sustainability. Challenges such as selecting an organic solvent or inorganic solution for regeneration, large energy requirements, complicated procedures for synthesizing these hydrogels, and eco-friendly nature (specifically synthetic hydrogels/aerogels) should be fully addressed. Thermosensitive hydrogels may be a good choice over traditional ones due to their ease of regeneration through temperature changes (requiring no harsh chemicals for regeneration) and high reusability. However, research in this field is still in its primary stage and requires an in-depth investigation.

Highly efficient synthetic or hybrid hydrogels have low natural biodegradability and high carbon footprints due to strong C–C, C–H, and C–N bonds, along with limited microbial degradation due to long chain lengths and high molecular weight.^138,139^ Biopolymer incorporation can enhance biodegradability with degradation rates depending on the biopolymer-to-synthetic polymer ratio. Inoculation of specific bacteria (e.g., *Pseudomonas*, *Rhodococcus*, *Phanerochaete chrysosporium*) into biodegradation media can accelerate breakdown of synthetic components. Lack of suitable enzymes also makes it challenging to break down the carbon chains of PAM hydrogels.

Embedding biobased polymers into synthetic hydrogels enhances the biodegradability of the resulting polymeric network because of the introduction of a new kind of carbon chain in the biopolymer backbone. However, the ratio of the biopolymer and synthetic polymer determines the degree of their biodegradability. The biopolymer parts degrade quickly; whereas synthetic portion may remain as it is for longer time.

Current hydrogel and aerogel research remains largely at the laboratory scale.^140^ Pilot-scale studies, cost reduction using biomaterials, and optimization of synthesis and operational parameters are needed for real-world PFAS removal, especially for both short- and long-chain PFAS. Life cycle assessments and environmental impact analyses are essential to evaluate long-term sustainability and ecological compatibility. Decision-making tools such as response surface methodology (tools used for optimizing the operational parameters), multicriteria decision analysis (for ranking hydrogels/aerogels adsorbents and treatment technologies taking into consideration their technical performance, cost, and LCA score), and LCA can guide the selection and commercialization of these materials.

Overall, hydrogels and aerogels hold significant potential as sustainable and efficient adsorbents for PFAS removal, combining tunable performance, regenerative capacity, and long-term practical utility in real-world water treatment applications.

## Supplementary Material



## Notes

Table of abbreviations, usage of different nonpolymeric PFAS in various fields, and references (PDF)
