# Supplementary material for: Mechanistic Insights and Design Strategies for Hydrogel/Aerogel Sorbents in Remediation of Per- and Polyfluoroalkyl Substances
Source: ACS Environ Au. 2025 Oct 20;6(1):1–20. doi: 10.1021/acsenvironau.5c00081 (PMC12828612; doi:10.1021/acsenvironau.5c00081)
Supplement: Supplementary file 1 [file vg5c00081_si_001.pdf]

Mechanistic Insights and Design Strategies for Hydrogel/Aerogel Sorbents in Per- and Polyfluoroalkyl Substances Remediation

Ashvinder Kumar[a], Manju K. Thakur[b], Phil Hart[c,d] and Vijay K. Thakur[a]*

*[a]Biorefining and Advanced Materials Research Center, SRUC, Kings Buildings, West Mains Road, Edinburgh, EH9 3JG, UK. *E-mail: Vijay.Thakur@sruc.ac.uk*

[b]Dept of Chemistry, RNT Govt College, Sarkaghat, Distt Mandi, HP, 175024, India

[c]The School of Water, Energy and Environment (SWEE), Cranfield University, MK43 0AL, UK

[d]Renewable and Sustainable Energy Research Centre, Technology Innovation Institute, P.O. Box 9639, Abu Dhabi, United Arab Emirates

Table of abbreviations

Name	Acronym		
2-(N-Ethylperfluorooctanesulfonamido)acetic acid	2N-EtFOSAA	Perfluoroalkyl acids	PFAAs
3-acrylamidopropyl)trimethylammonium chloride	DMAPAQ	Perfluoroalkyl iodides	PFAIs
3-aminopropyltriethoxysilane	APTES	Perfluoroalkylcarboxylic Acids	PFCA
6:2-Fluorotelomersulfonic acid	6:2 FTSA	Perfluoroalkylsulfonic acid	PFSA
Activated carbon	AC	Perfluorobutanesulfonic acid	PFBS
Amino-functionalized graphene oxide	AGO	Perfluorobutanoic acid	PFBA
Carbon dots	CDs	Perfluorodecanoic acid	PFDA
Chitosan	CS	Perfluoroheptanoic acid	PFHpA
Graphene oxide	GO	Perfluorohexanesulfonic acid	PFHxS
Ionic fluorogels	IF	Perfluorohexanoic acid	PFHxA
kappa-carrageenan	kC	Perfluorononanoic acid	PFNA
Lifecycle cost analysis	LCA	Perfluorooctanesulfonic acid	PFOS
Lifetime Health Advisory	LHA	Perfluorooctanoic acid	PFOA
Lower critical solution temperature	LCST	Perfluoropentanoic acid	PFPeA
Methyltrimethoxysilane	MTMS	Perfluoropolyethers	PFPEs
N,N'-Methylenebisacrylamide	NNMBA	Perfluoroundecanoic acid	PFUnA
N-[3-(dimethylamino)propyl]acrylamide, methyl chloride quaternary,	DMAPAA-Q	Persistent organic pollutants	POPs
Natural organic matters	NOMs	Point of zero charge	pHzpc
N-isopropylacrylamide	NIPAm	Porous activated carbon	PAC
P123 dimethacrylate	PDM	Sodium alginate	SA
Per and polyfluoroalkyl substances	PFAS	Trianglamine	3D-SH

Perfluoro-2-methyl-3-oxahexanoic acid	HFPO-DA or GenX	U.S. Environmental Protection Agency	USEPA
perfluoroalkane sulfonyl fluoride	PASF	Wastewater treatment plants	WWTPs

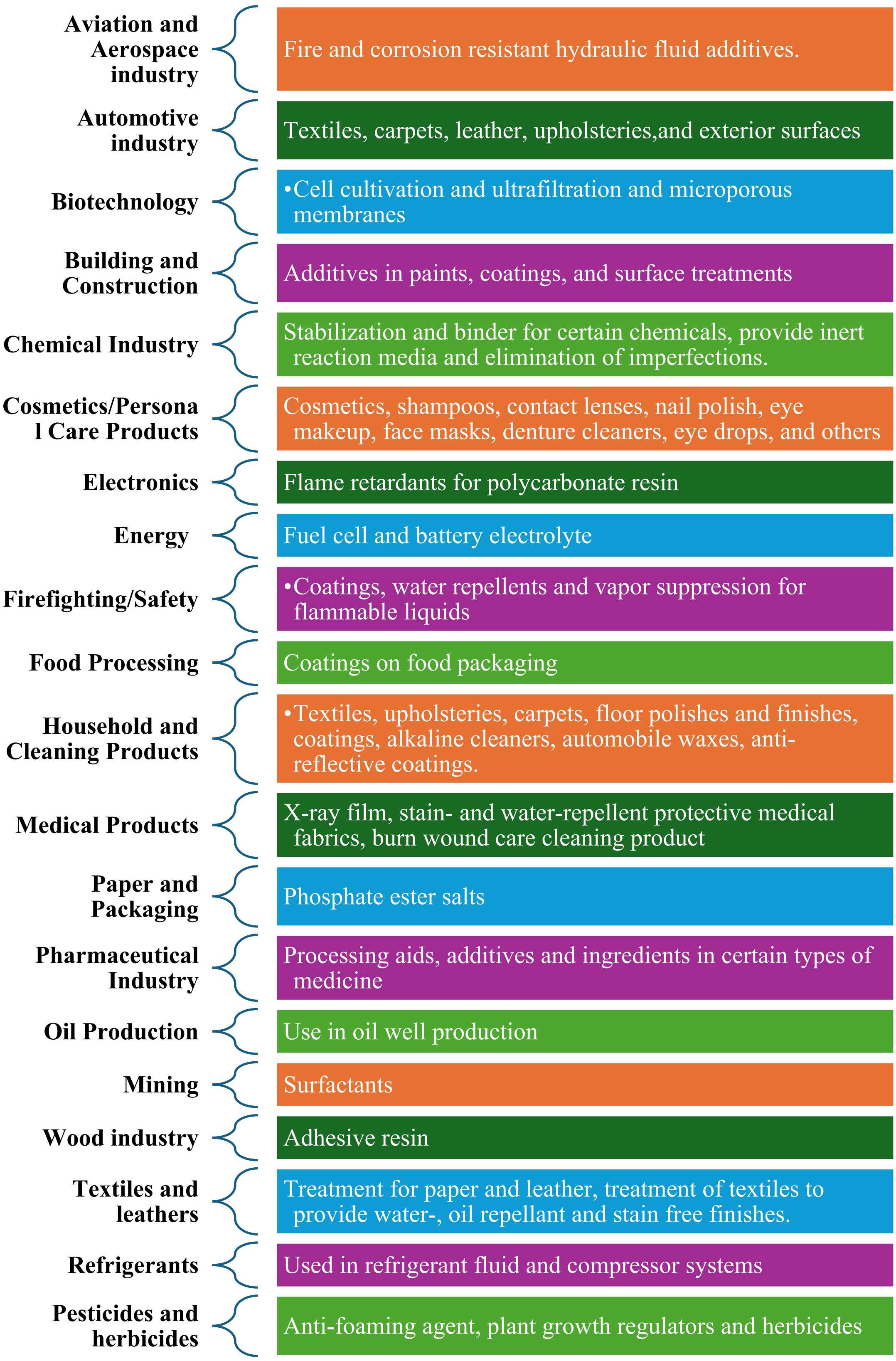

Figure S1. Showing the usage of different non-polymeric PFAS in various fields [1].

“Figure redrawn from Ref. [1] under CC BY-NC 3.0)”.

Reference

(1) Glüge, J.; Scheringer, M.; Cousins, I. T.; DeWitt, J. C.; Goldenman, G.; Herzke, D.; Lohmann, R.; Ng, C. A.; Trier, X.; Wang, Z. An Overview of the Uses of Per-and Polyfluoroalkyl Substances (PFAS). *Environ. Sci. Process. Impacts* **2020**, *22* (12), 2345–2373.
